# Cuproptosis-related ceRNA axis triggers cell proliferation and cell cycle through CBX2 in lung adenocarcinoma

**DOI:** 10.1186/s12890-024-02887-0

**Published:** 2024-02-14

**Authors:** Jiang Wu, Guang Fu, Chao Luo, Liang Chen, Quanxing Liu

**Affiliations:** Department of Thoracic Surgery, Xinqiao Hospital, Army Medical University, 400037 Chongqing, China

**Keywords:** LUAD, ceRNA, Cell proliferation

## Abstract

**Background:**

Lung adenocarcinoma (LUAD) has high morbidity and mortality. Despite substantial advances in treatment, the prognosis of patients with LUAD remains unfavorable. The ceRNA axis has been reported to play an important role in the pathogenesis of LUAD. In addition, cuproptosis is considered an important factor in tumorigenesis. The expression of CBX2 has been associated with the development of multiple tumors, including LUAD. However, the precise molecular mechanisms through which the cuproptosis-related ceRNA network regulates CBX2 remain unclear.

**Methods:**

The DEGs between tumor and normal samples of LUAD were identified in TCGA database. The “ConsensusClusterPlus” R package was used to perform consensus clustering based on the mRNA expression matrix and cuproptosis-related gene expression profile. Then, LASSO-COX regression analysis was performed to identify potential prognostic biomarkers associated with cuproptosis, and the ceRNA network was constructed. Finally, the mechanisms of ceRNA in LUAD was studied by cell experiments.

**Results:**

In this study, the AC144450.1/miR-424-5p axis was found to promote the progression of LUAD by acting on CBX2. The expression of AC144450.1 and miR-424-5p can be altered to regulate CBX2 and is correlated with cell proliferation and cell cycle of LUAD. Mechanistically, AC144450.1 affects the expression of CBX2 by acting as the ceRNA of miR-424-5p. In addition, a cuproptosis-related model were constructed in this study to predict the prognosis of LUAD.

**Conclusions:**

This study is the first to demonstrate that the AC144450.1/miR-424-5p/CBX2 axis is involved in LUAD progression and may serve as a novel target for its diagnosis and treatment.

**Supplementary Information:**

The online version contains supplementary material available at 10.1186/s12890-024-02887-0.

## Background

Lung adenocarcinoma (LUAD), particularly in advanced stages, is highly malignant and has an unfavorable prognosis [1]. Despite substantial advancements in treatment, the 5-year survival rate of patients with LUAD is 12–15% [2]. Currently available diagnostic methods, such as imaging, biopsy, and molecular testing, have limitations, including the high likelihood of false-negative results and the inability to detect early-stage LUAD [3]. In contrast, novel diagnostic techniques such as next-generation sequencing and companion diagnostics have demonstrated great potential in identifying genomic alterations and biomarkers that can facilitate early detection and prompt personalized treatment [4]. Additionally, novel techniques for detecting early-stage cancer and understanding its progression are under development [5]. Traditional therapeutic strategies such as surgery, chemotherapy, and radiation therapy have disadvantages, including side effects, drug resistance, and the inability to treat advanced-stage LUAD [6]. However, novel therapies such as immunotherapy and targeted therapy, have shown beneficial effects in the treatment of LUAD. Therefore, developing more effective diagnostic and treatment methods is necessary for improving the prognosis of patients with LUAD.

MicroRNAs (miRNAs) modulate gene expression by binding to the 3´-untranslated region (UTR) of target mRNAs. They are small non-coding RNA (ncRNAs) that can either degrade the target mRNA or inhibit translation [7]. miRNAs play a crucial role in regulating the cell cycle, metastasis, angiogenesis, metabolism, and apoptosis, thus influencing tumorigenesis [8]. In lung cancer, miRNAs act as both tumor suppressors and oncomirs, regulating the expression of target mRNAs to influence tumor cell proliferation, angiogenesis, and metastasis [9]. miR-424-5p, a distinct miRNA variant, is involved in regulating gene expression and the progression of several cancer types [10]. For instance, it can hinder the proliferative, migratory, and invasive capabilities of lung cancer cells [11]. Long non-coding RNAs (lncRNAs) are RNA transcripts that exceed 200 nucleotides in length and lack protein-coding ability but play a pivotal role in gene regulation [12]. lncRNAs can interact with DNA, RNA, and proteins to regulate chromatin structure, transcription, post-transcriptional processes, and translation [13]. They act as molecular decoys for miRNAs and regulate splicing, mRNA decay, translation, and protein stability [14]. In addition, they are involved in the development of various types of cancers, including LUAD [15]. For example, the lncRNA MALAT1 induces tumor cell proliferation and metastasis by regulating downstream genes in LUAD [16], and the lncRNA HOTAIR induces tumor cell proliferation and invasion by regulating downstream genes in LUAD [17]. lncRNAs can be used as biomarkers for the diagnosis and treatment of LUAD. The lncRNA AC144450.1, also known as ENSG00000228613, is involved in various biological processes, including cancer development [18]. However, further studies are warranted to examine the precise role of AC144450.1 in biological processes and assess its therapeutic potential. Competitive endogenous RNAs (ceRNAs) is a interaction between RNAs crucial in developing neoplasms [19]. It involves lncRNA acting as an endogenous sponge to effect mRNA expression via sinking miRNA [20]. ceRNAs have been reported to play a vital role in the initiation and development of LUAD [21]. lncRNAs and miRNAs that are inversely related to each other may serve as biomarkers for the diagnosis, prognosis, and treatment of cancer [22].

The presence of excessive copper can induce cell death. The mechanisms underlying copper-induced cell death remained elusive until the identification of cuproptosis [23]. Cuproptosis is a programmed cell death mode induced by copper accumulation and proteotoxic stress [24]. Cuproptosis has been associated with various cancers, including LUAD [25], and cellular metabolism. Certain cancer types often exhibit elevated levels of aerobic respiration [23]. Numerous studies have investigated the relationship between cuproptosis-related genes and diverse tumor characteristics [26]. Some cancers, such as breast cancer and leukemia, have been associated with cuproptosis [27]. Studies have reported the development of risk models based on cuproptosis-related genes to predict prognosis and assess immune cell infiltration in LUAD [28, 29]. In addition, studies have revealed cuproptosis-related genes that can serve as therapeutic targets for LUAD [30].

CBX2 is a gene responsible for encoding an element of the polycomb multiprotein complex that is essential for preserving the transcriptionally repressive state of numerous genes [31]. CBX2 functions as both a transcriptional repressor and activator, thus playing an important role in regulating gene expression, cell differentiation, and cell fate commitment [32]. It has been demonstrated to be upregulated in various cancers, such as breast and colorectal cancers [33]. It is markedly upregulated in LUAD and is associated with an unfavorable prognosis [34]. In addition, it promotes tumor growth and metastasis by regulating gene expression in LUAD [35]. Altogether, CBX2 has emerged as a prospective therapeutic target for LUAD [35]. However, whether ncRNAs regulate CBX2 in LUAD and the underlying regulatory mechanisms remain unclear. Therefore, we attempt to investigate the pontential mechanisms of the cuproptosis-related ceRNA network regulating CBX2.

In this study, AC144450.1 was identified as a ceRNA of miR-424-5p and was found to regulate the cuproptosis-related gene CBX2 in LUAD. Initially, we investigated the regulatory effects of AC144450.1, miR-424-5p, and CBX2 on the malignant progression of LUAD in TCGA database. Subsequently, we predicted and validated the interaction between AC144450.1 and miR-424-5p, demonstrating the role of AC144450.1 as a molecular sponge that regulates CBX2. Mechanistically, the function of miR-424-5p in the malignant progression of LUAD relies on CBX2 to some extent. Altogether, this study introduces a novel and effective approach to diagnosing and treating LUAD.

## Methods

### Data collection

The RNA-seq data and clinical information of patients with LUAD were extracted from TCGA database. The miRNA microarray dataset GSE62182 was downloaded from the GEO database. Patients with other malignancies and those with incomplete clinical information were excluded.

### Differential gene expression analysis

The “limma” package was used to identify differentially expressed genes (DEGs) between lung cancer samples and corresponding adjacent normal tissue samples. Significant DEGs were defined by adjusted *P*-values of < 0.05 and|fold change (FC)| values of > 1.0.

### Functional enrichment analysis

The “clusterProfiler” software package was used to implement GO functional annotation and KEGG pathway enrichment analyses. Significantly enriched pathways were defined by false discovery rates (FDRs) < 0.05. Bubble maps were drawn to visualize the results of enrichment analyses, whereas a chord diagram was drawn using the “circlize” R package to visualize mRNAs in each pathway.

### Consistent cluster analysis

The “ConsensusClusterPlus” R package was used to perform consensus clustering based on the mRNA expression matrix and cuproptosis-related gene expression profile. All samples were divided into k groups (k = 2–9). The optimal number of clusters was determined using two methods: the consensus matrix and the cumulative distribution function (CDF). In addition, Kaplan-Meier curves were plotted to compare survival outcomes between subgroups.

### Construction of the lncRNA–microRNA–mRNA network

The StarBase and TargetScan databases were used to predict interactions between lncRNAs and mRNAs based on sequence complementarity. The miRDB, miRTarBase, and TargetScan databases were used to predict mRNAs regulated by miRNAs. These databases use algorithms to predict potential miRNA targets based on sequence complementarity and experimental evidence. Finally, a lncRNA-miRNA-mRNA regulatory network based on ceRNAs was constructed by integrating the predicted lncRNA-miRNA and miRNA-mRNA pairs, and the Cytoscape (version 3.7.2) software was used to visualize the ceRNA network.

### Analysis of prognostic biomarkers associated with cuproptosis

LASSO-COX regression analysis was performed to identify potential prognostic biomarkers associated with cuproptosis. The risk score of each patient was calculated based on the prognostic model, and the patients were divided into high- and low-risk groups. The lower the risk score, the lower the recurrence risk. Kaplan-Meier curves were plotted to compare survival between the two risk groups. In addition, receiver operating characteristic (ROC) curves were plotted using the “survival ROC” R package.

### Cell culture

The human lung cancer cell line A549 and HCC827 used in this study were purchased from the National Authenticated Cell Culture Collection in Shanghai, China. A549 cells were cultured in DMEM supplemented with 10% fetal bovine serum and 1% penicillin–streptomycin (to prevent bacterial contamination) (Gibco, USA). HCC827 were cultured in RPMI-1640 supplemented with 10% fetal bovine serum and 1% penicillin–streptomycin (to prevent bacterial contamination) (Gibco, USA).

### Cell transfection

Transfection was performed using Lipofectamine 2000 (Invitrogen) according to the manufacturer’s instructions. Cells were seeded in 6-well plates at an optimal density and transfected with plasmids using Lipofectamine 2000 and Opti-MEM after 24 h. Empty plasmids and plasmids encoding an miR-424-5p mimic and an miR-424-5p inhibitor were procured from GenePharma (Shanghai, China). After 48 h of transfection, the cells were harvested for subsequent experiments.

### RNA extraction and qRT-PCR

Total RNA was extracted from cells and reverse transcribed into cDNA using a reverse transcription kit. The synthesized cDNA was amplified via qPCR using the SYBR Premix Ex Taq kit. The relative RNA expression was quantified using the 2-ΔΔCt method. All reagents used for qRT-PCR were purchased from TaKaRa, Japan.

### Protein extraction and western blotting

Transfected cells were harvested and lysed in RIPA buffer (Beyotime Biotechnology, China) containing a protease inhibitor mixture. The extracted proteins (in equal amounts) were separated via SDS-PAGE and transferred to PVDF membranes, followed by incubation with primary and secondary antibodies.

### CCK8 assay

To evaluate cell viability, cells were seeded in a 96-well plate and cultured until they reached 70–80% confluence. Thereafter, 10 μL of the CCK-8 reagent was added to each well, and the cells were incubated for 2 h. Absorbance was measured at 450 nm using a microplate reader, and the percentage of cell viability was calculated relative to the control group.

### Cell cycle

Cells were harvested after transfection, washed twice with precooled PBS, and digested with pancreatin without EDTA. The cells were fixed with 70% ethanol overnight, washed twice with PBS, and stained with 500 μL of PI/RNaSeA solution for 30 min at room temperature. Subsequently, the cell cycle was analyzed on an FACSCalibur flow cytometer (BD).

### Luciferase assay

The promoter region of the target gene was cloned into a pGL3-based vector upstream of the firefly luciferase gene. The 3´-untranslated region (UTR) of the target gene downstream of the Renilla luciferase gene was cloned into the pRL-TK vector. Cells were seeded in 24-well plates and cultured until they reached 70–80% confluence. The cells were co-transfected with reporter plasmids and either wild-type (WT) or mutant (MUT) lncRNA AC144450.1 using transfection reagents. Transfected cells were harvested after 24–48 h and washed with PBS. Cell lysates were collected, and the activity of firefly and Renilla luciferases was measured on a photometer.

### Statistical analysis

The GraphPad Prism 8 software was used for statistical analysis, and all data were expressed as the mean ± standard deviation. The two-tailed Student’s t-test was used to compare the data of two groups. Survival curves were plotted using the Kaplan-Meier plotter and compared using the log-rank (Mantel-Cox) test. Statistical significance was denoted as follows: *, *P* < 0.05; **, *P* < 0.01; ***, *P* < 0.001.

## Results

### Identification of lncRNAs, miRNAs, and mRNAs related to the progression of lung cancer

**Fig. 1:**
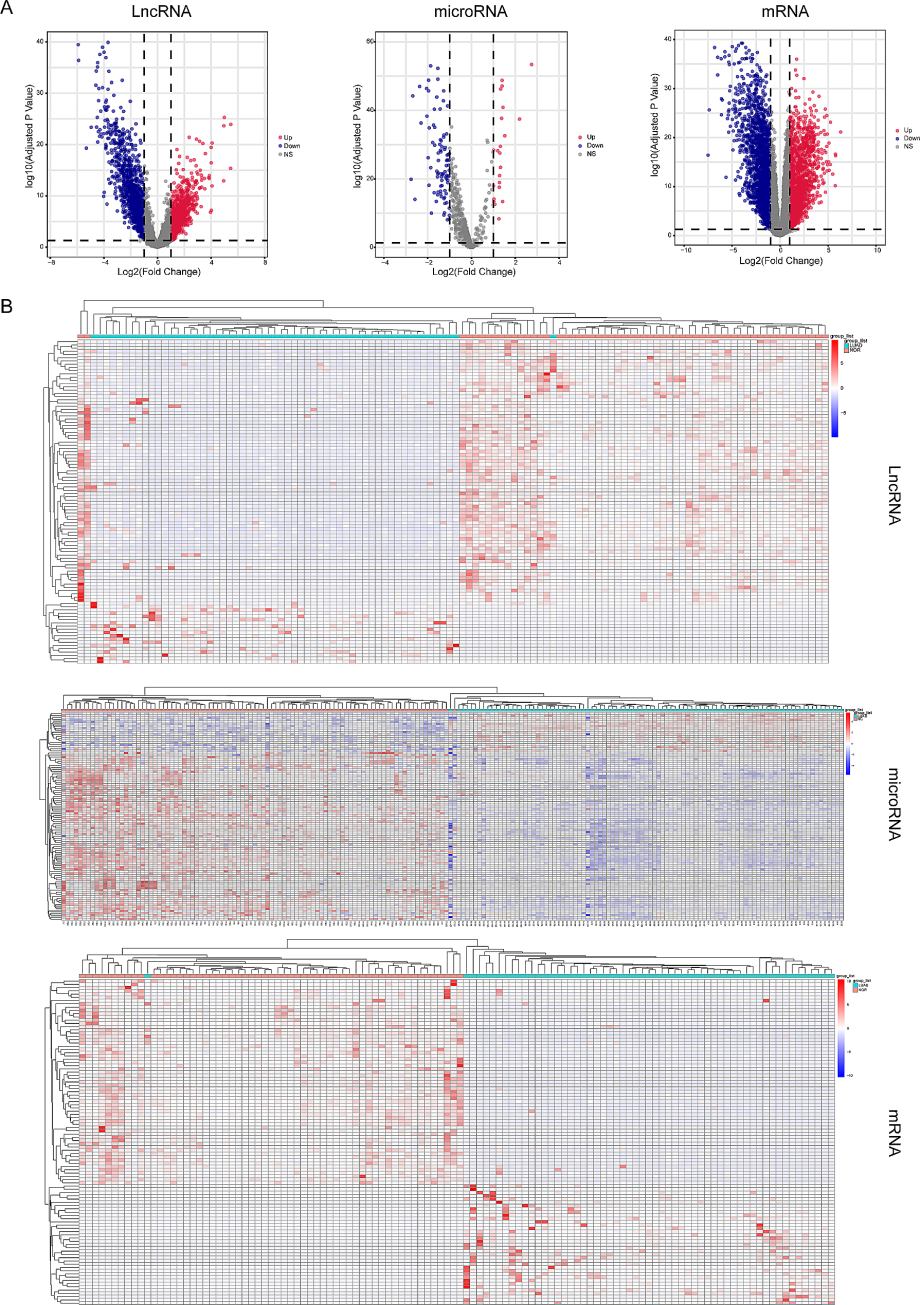
Screening the lncRNA, microRNA and mRNA related to lung cancer progression. (**A**) Volcano plots of differential expression analysis of mRNAs, lncRNAs and miRNAs. (**B**) Heat map of differential expression analysis of mRNAs, lncRNAs and miRNAs

Differentially expressed lncRNAs and mRNAs were identified between 58 matched tumor and normal samples in TCGA-LUAD dataset. A volcano map was constructed to identify significant DEGs based on *P*-values of < 0.05 and log_2_FC values of ≥ 1 or ≤ -1 after adjusting for multiple comparisons. The expression of both mRNAs and lncRNAs was significantly different between tumor and normal tissues. Specifically, 1980 upregulated and 2362 downregulated mRNAs as well as 1184 upregulated and 1476 downregulated lncRNAs were identified in lung cancer tissues (Fig. 1A-B and Supplementary Fig. 1A-C). Furthermore, differentially expressed miRNAs were screened in the GSE62182 dataset using GEO2R. A total of 24 miRNAs were upregulated and 89 miRNAs were downregulated in lung cancer tissues. These DEGs were visualized on heatmaps generated using the ggplot2 R package. Tumor and non-tumor tissues were separately clustered based on their respective lncRNA, miRNA, and mRNA expression patterns.

### Pathway enrichment analysis of genes related to the progression of lung cancer

**Fig. 2:**
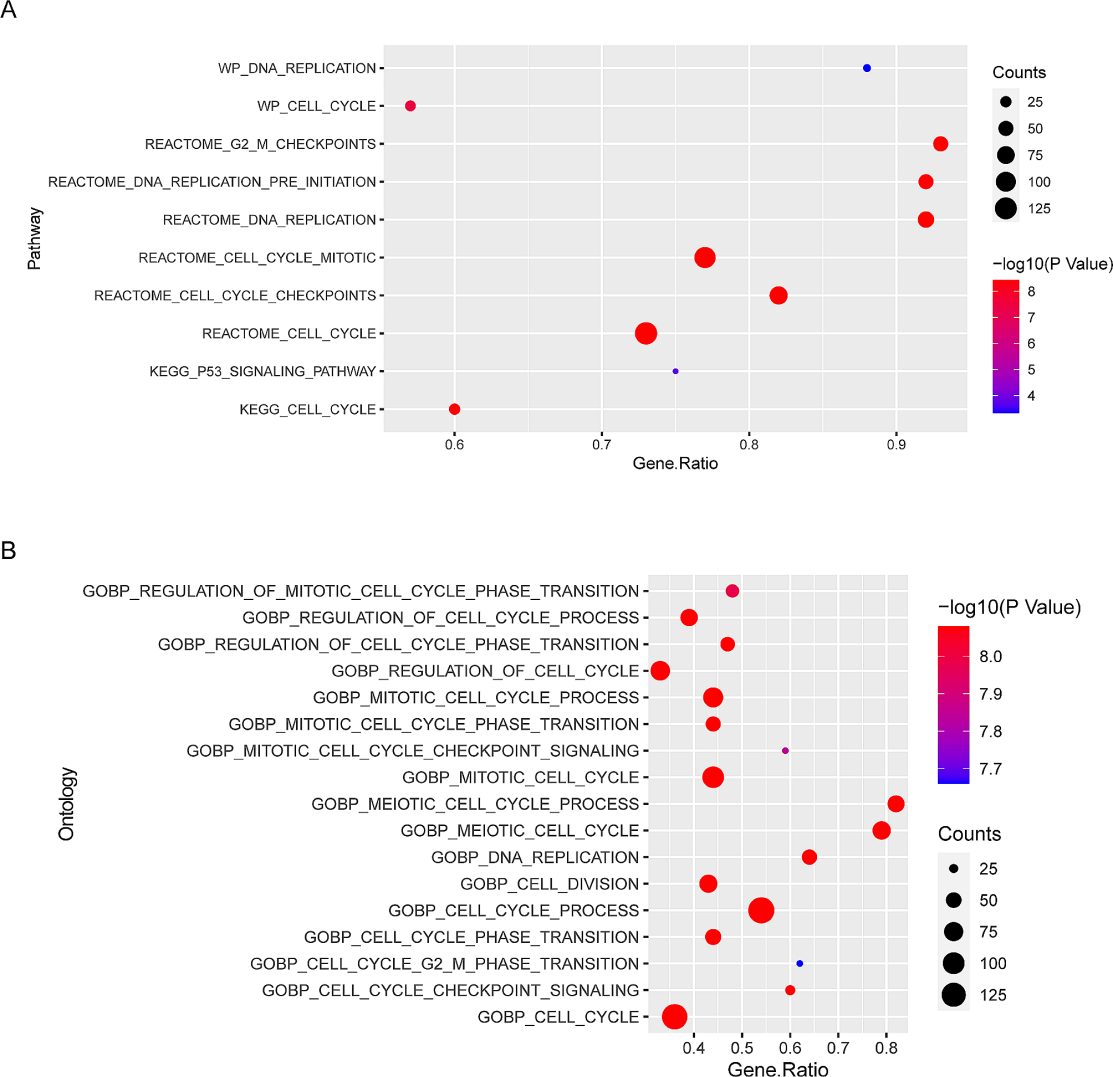
Pathway enrichment of genes related to lung cancer progression. (**A**) KEGG enrichment analysis of regulated mRNAs. (**B**) GO enrichment analysis of regulated mRNAs

Gene set enrichment analysis (GSEA) was performed to examine the significance of the identified DEGs in lung cancer. KEGG analysis revealed that the DEGs were mainly enriched in pathways related to cell proliferation and DNA replication (Fig. 2A and Supplementary Fig. 2A-C). Furthermore, GO analysis revealed that the DEGs were primarily involved in cell proliferation, cell cycle transition, activation of cell cycle-related signaling pathways, and DNA replication (Fig. 2B and Supplementary Fig. 2D-F). Altogether, the results indicated that genes regulating the malignant progression of lung cancer were involved in cell proliferation and cell cycle.

### Consensus cluster analysis of cuproptosis-related genes

**Fig. 3:**
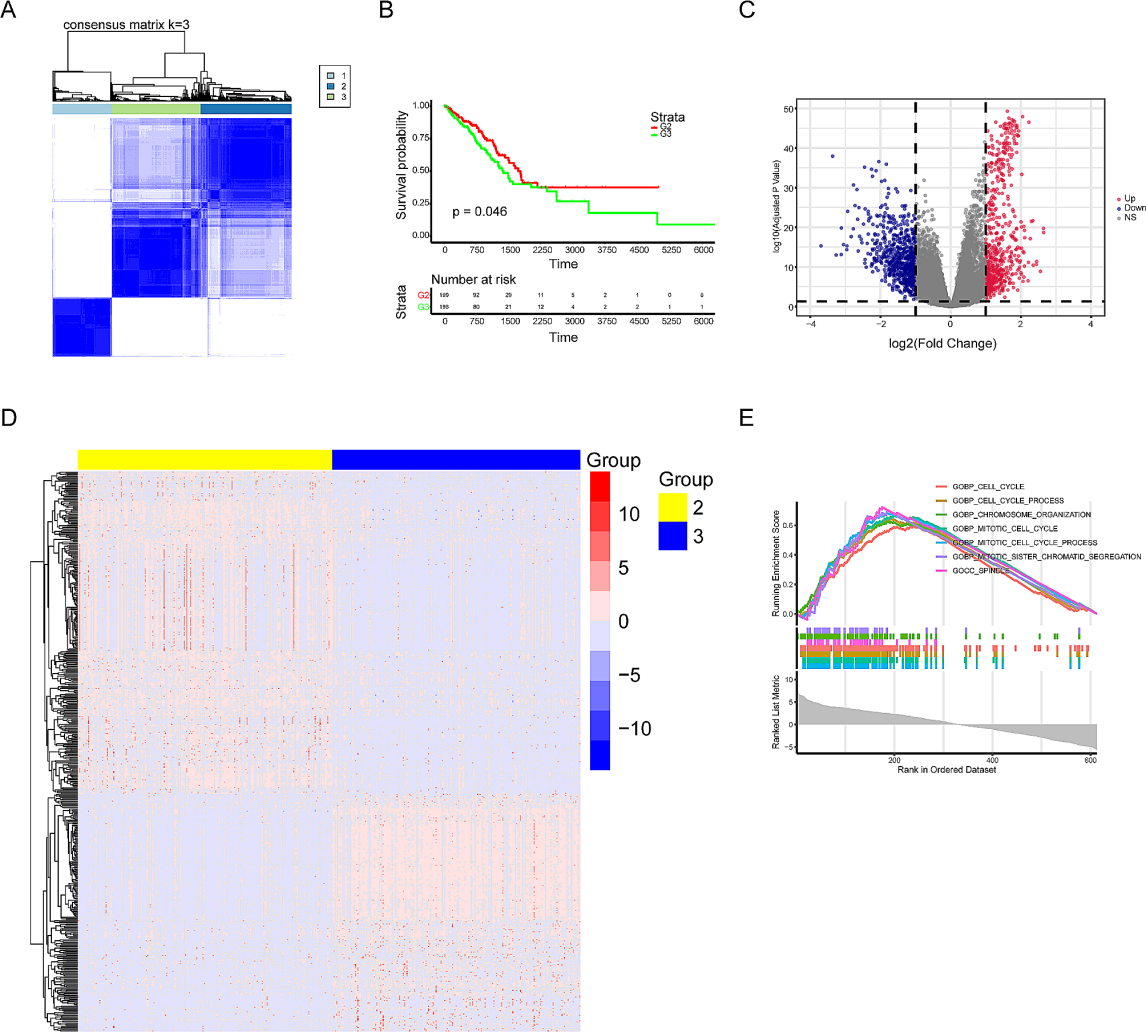
Consensus cluster analysis of cuproptosis related genes. (**A**) Consensus clustering heatmap of genes associated with cuproptosis with k = 3. (**B**) Kaplan-Meier survival curve between cluster 2 and cluster 3. (**C**) The volcano map showed the differential genes of the two clusters. (**D**) Heatmap of differential genes. (**E**) Pathway enrichment for cuproptosis-related differentially expressed genes obtained by GSEA

To examine the potential role of cuproptosis in lung cancer, we performed cluster analysis to evaluate the expression level of cuproptosis of lung cancer in TCGA database, The results revealed that there are three groups of differential expression levels of cuproptosis (Fig. 3A and Supplementary Fig. 3A-B). The group 2 and 3 were choosed for further differential gene analysis according to the survival result (Fig. 3B). A total of 1325 DEGs were identified, including 611 upregulated and 714 downregulated genes (Fig. 3C-D and Supplementary Fig. 3C). GSEA revealed that these DEGs were primarily involved in pathways related to cell proliferation, cell cycle, and other cellular processes (Fig. 3E and Supplementary Fig. 3D).

### lncRNA AC144450.1/miR-424-5p/CBX2 may be an important ceRNA axis in the regulation of lung cancer

**Fig. 4:**
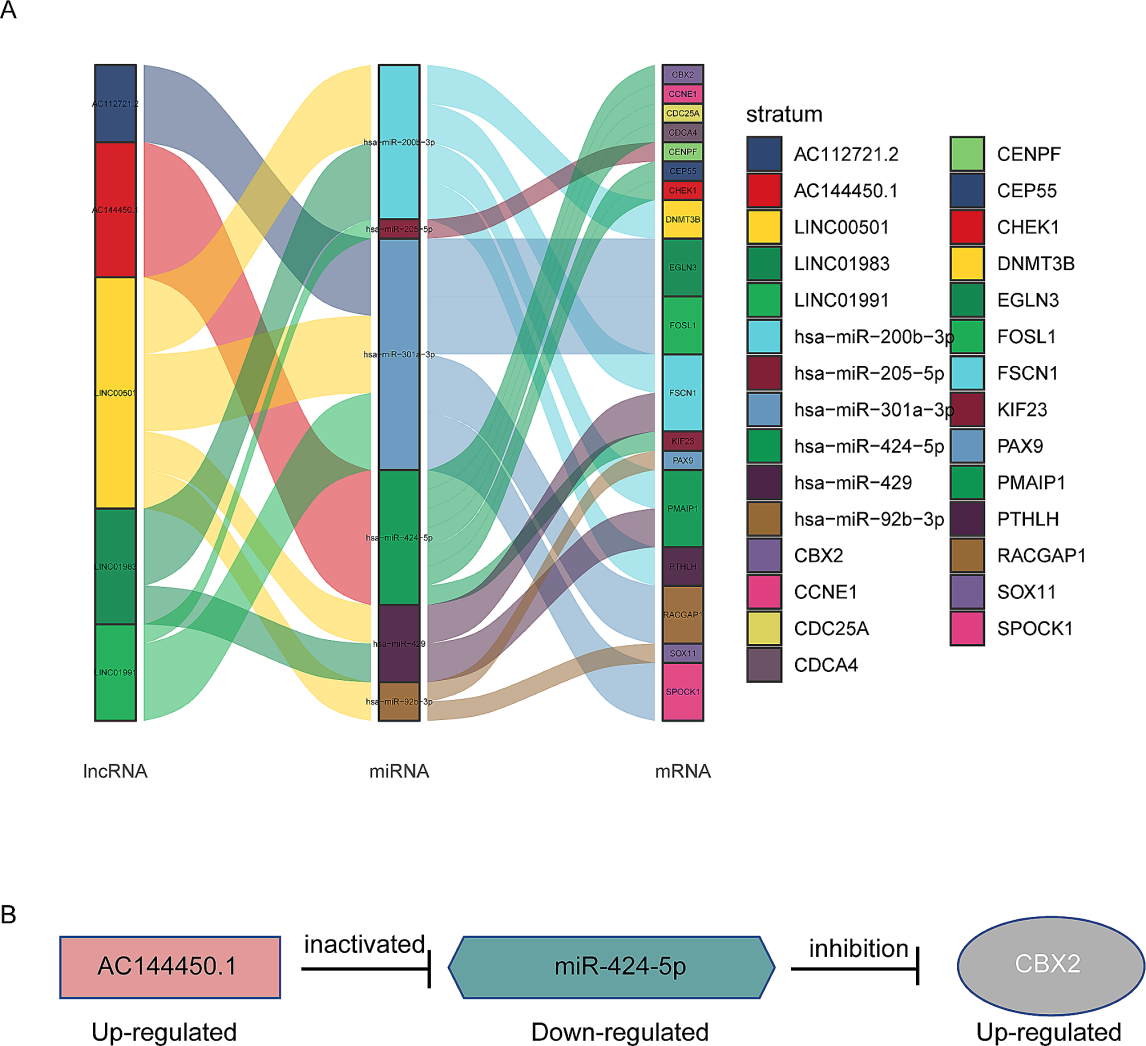
LncRNA-AC144450.1/miR-424-5p/CBX2 may be an important ceRNA axis in the regulation of lung cancer. (**A**) Sankey diagram for the ceRNA network in lung cancer. Each rectangle represents a gene, and the connection degree of each gene is visualized based on the size of the rectangle. (**B**) The **c**eRNA regulatory network

To gain deeper insights into the regulatory role of lncRNAs in mediating miRNA-mRNA interactions in lung cancer, a ceRNA network was constructed including the differentially expressed lncRNAs, miRNAs, and mRNAs involved in regulating the malignant progression of lung cancer. According to the ceRNA hypothesis, a positive regulatory relationship exists between lncRNAs and mRNAs, whereas a negative regulatory relationship exists between miRNAs and mRNAs. Eventually, 5 lncRNAs, 6 miRNAs, 18 mRNAs, and 34 interaction axes were identified, which were visualized using the Cytoscape (version 3.9.0) software (Fig. 4A and Supplementary Fig. 4). AC144450.1 and miR-424-5p were identified as the lncRNA and miRNA, respectively, involved in the ceRNA axis regulating CBX2 (Fig. 4B). The previous studies found that CBX2 acts as a transcription factor regulating numerous cell functions, which means CBX2 exerts important role in cancer development. Therefore, the AC144450.1/miR-424-5p/CBX2 axis was selected for further experiments.

### lncRNA AC144450.1/miR-424-5p/CBX2 axis may serve as a prognostic biomarker for lung cancer

**Fig. 5:**
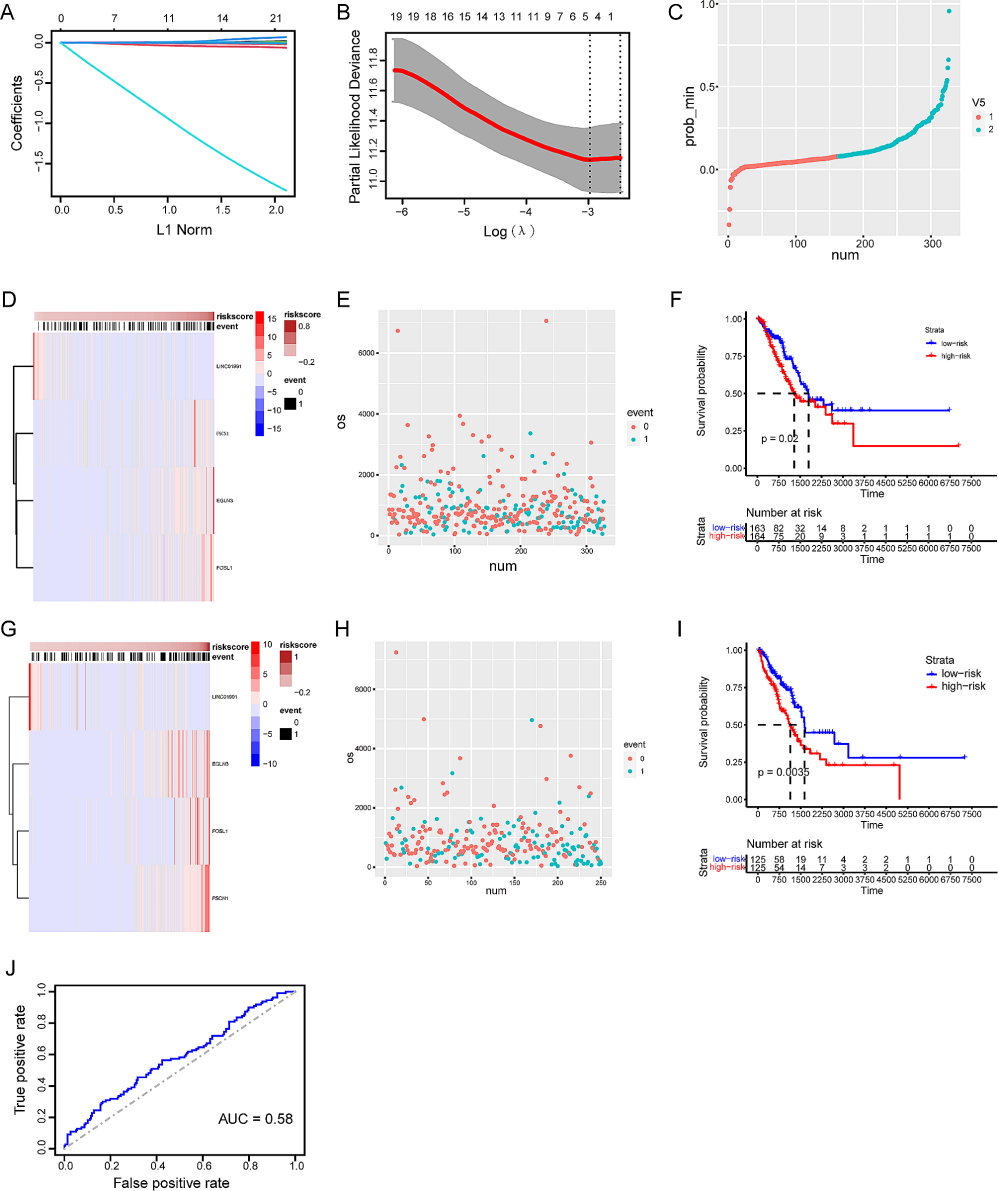
LncRNA-AC144450.1/miR-424-5p/CBX2 axis could be a better biomarker for prognosis of lung cancer. (**A**) LASSO regression coefficient profile of the intersection genes. (**B**) LASSO deviance profile of the intersection genes. (**C**) Distribution of risk score between low and high-risk groups in the training cohort. (**D**) The heatmap based on the risk score in the training cohort. (**E**) Survival status plot of the training cohort. (**F**) Survival curves for the two groups in the training cohort. (**G**) The heatmap based on the risk score in the validation cohort. (**H**) Survival status plot of the validation cohort. (**I**) Survival curves for the two groups in the validation cohort. (**J**) ROC curves based on the risk score in the training cohort

Previous studies have demonstrated that AC144450.1, miR-424-5p, and CBX2 are potential prognostic biomarkers for lung cancer. Because the malignant progression of lung cancer is the result of the collective regulatory effects of multiple gene networks, using a single gene as a diagnostic marker has limitations. To investigate the potential prognostic value of the identified biomarkers, LASSO regression analysis (Fig. 5A-E) was performed to construct a cuproptosis-related prognostic signature incorporating the three biomarkers. According to this signature, the risk scores of patients with LUAD were calculated using the following formula: LINC01991 * (-0.124) + FSCN1 * 0.0007 + EGLN3 * 0.0014 + FOSL13 * 0.0006. The patients were divided into high- and low-risk groups based on the median risk score. The risk scores were significantly associated with survival in both groups. The prognosis of high-risk patients was poorer than that of low-risk patients, and the AUC value of the polygenic model was higher than that of the single-gene model (Fig. 5F and J, Supplementary Fig. 5A-D). Subsequently, the high predictive performance of the prognostic model was verified in the validation set (Fig. 5G-I). Altogether, the prognostic model constructed based on AC144450.1, miR-424-5p, and CBX2 detected lung cancer and predicted the prognosis more accurately.

### lncRNA AC144450.1/miR-424-5p/CBX2 regulates the malignant progression of lung cancer

**Fig. 6:**
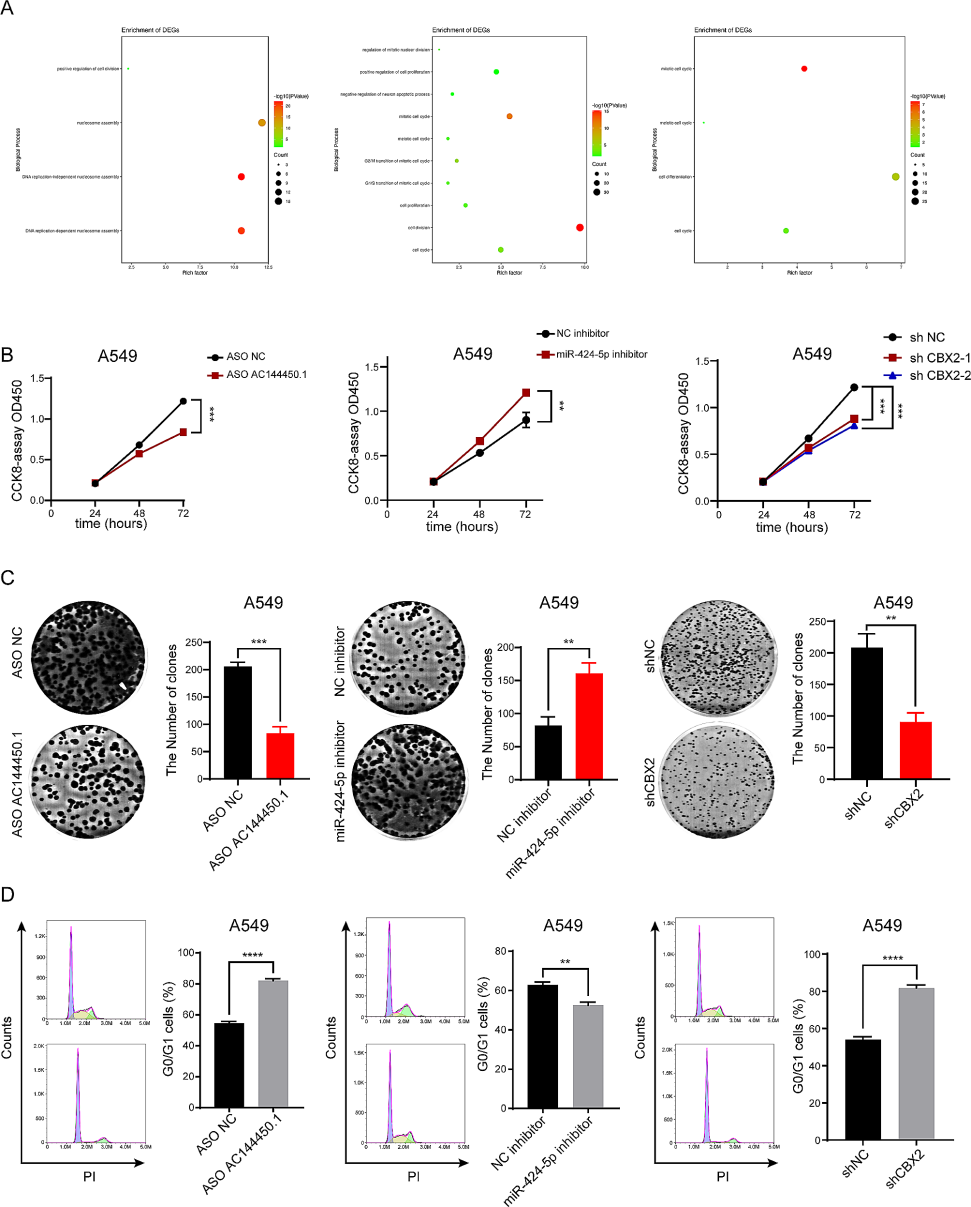
LncRNA-AC144450.1/miR-424-5p/CBX2 proteins regulated the malignant progression of lung cancer. (**A**) GSEA enrichment map. (**B**-**D**) CCK-8, cell clone and cell cycle assay showed that AC144450.1, miR-424-5p or CBX2 regulate A549 cells malignant progression

According to the abovementioned results, the AC144450.1/miR-424-5p/CBX2 axis functions as the ceRNA network that regulates the occurrence and development of lung cancer. However, the precise role of AC144450.1, miR-424-5p, and CBX2 in the malignant progression of lung cancer remains unclear. Therefore, we divided the samples into high- and low-expression groups based on the median expression of the three factors and performed differential expression and enrichment analyses for each group. The results revealed that miRNA- and mRNA-regulated genes were enriched in pathways associated with cell proliferation and cell cycle (Fig. 6A and Supplementary Fig. 6A-B). Besides, we analysed the differentially expressed levels of the three genes in EGFR wild type and mutation type groups in TCGA LUAD patients, the results showed that CBX2 is significantly higher expression comparing with the EGFR mutation group (Supplementary Fig. 6C). Next, the expression of AC144450.1, miR-424-5p, and CBX2 was individually knocked down in lung cancer cells to examine the regulatory effects of the ceRNA network on cell proliferation and cell cycle. The results of CCK8 assay, colony formation assay, and cell cycle analysis revealed that the knockdown of each gene led to a significant reduction in cell proliferation and altered the cell cycle of lung cancer cells (Fig. 6B-D and Supplementary Fig. 7A-C). These results were consistent with those of enrichment analyses. Altogether, the results revealed that AC144450.1, miR-424-5p, and CBX2 individually regulated the malignant progression of lung cancer.

### lncRNA AC144450.1 and mir-424-5p regulate the CBX2 protein in lung cancer

**Fig. 7:**
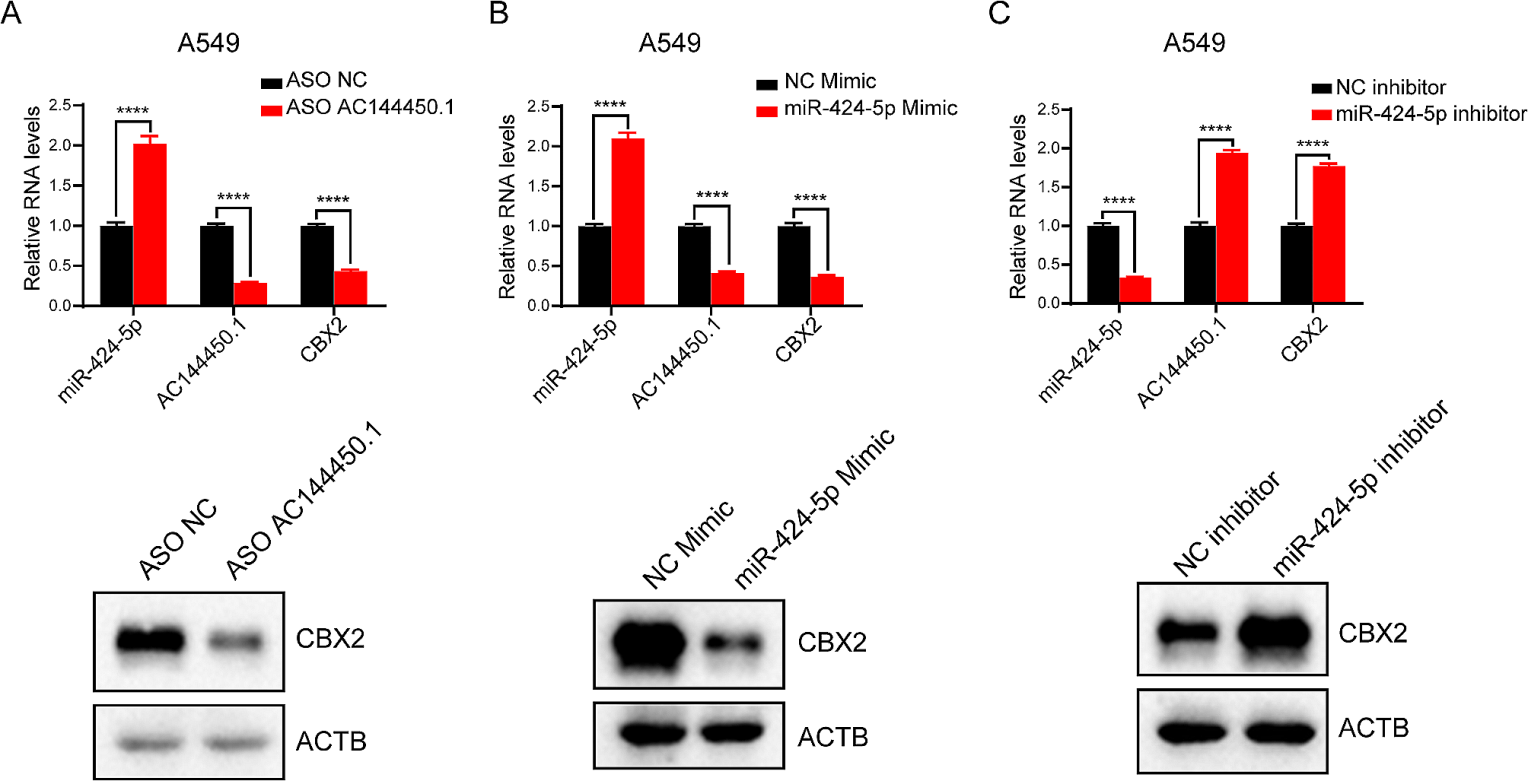
LncRNA-AC144450.1 and miR-424-5p could regulate CBX2 protein in lung cancer. (**A**) RT-qPCR assays and western blot were used to detect the expression of AC144450.1, miR-424-5p and CBX2 in A549 cells treated with AC144450.1 ASO. (**B**) RT-qPCR assays and western blot were used to detect the expression of AC144450.1, miR-424-5p and CBX2 in A549 cells treated with miR-424-5p mimic. (**C**) RT-qPCR assays and western blot were used to detect the expression of AC144450.1, miR-424-5p and CBX2 in A549 cells treated with miR-424-5p inhibitor

To examine the relationship among AC144450.1, miR-424-5p, and CBX2 proteins, we used antisense oligonucleotides (ASOs) to knock down AC144450.1 and assessed changes in the expression of miR-424-5p, CBX2 using western blotting. The results revealed that AC144450.1 knockdown decreased the expression of AC144450.1, resulting in subsequent downregulation of CBX2 and upregulation of miR-424-5p (Fig. 7A and Supplementary Fig. 8A). Furthermore, miR-424-5p mimic and inhibitor were used to overexpress and inhibit it, respectively, and changes in the expression of AC144450.1 and CBX2 protein were detected. The results revealed that the signaling pathway regulated by AC144450.1 and CBX2 was negatively correlated with the expression of miR-424-5p (Fig. 7B-C and Supplementary Fig. 8B-C). Altogether, these results indicate that AC144450.1 and miR-424-5p regulate the CBX2 protein in lung cancer, which is consistent with the ceRNA hypothesis.

### AC144450.1 as the ceRNA of miR-424-5p regulates the CBX2 protein and affects the malignant progression of lung cancer

**Fig. 8:**
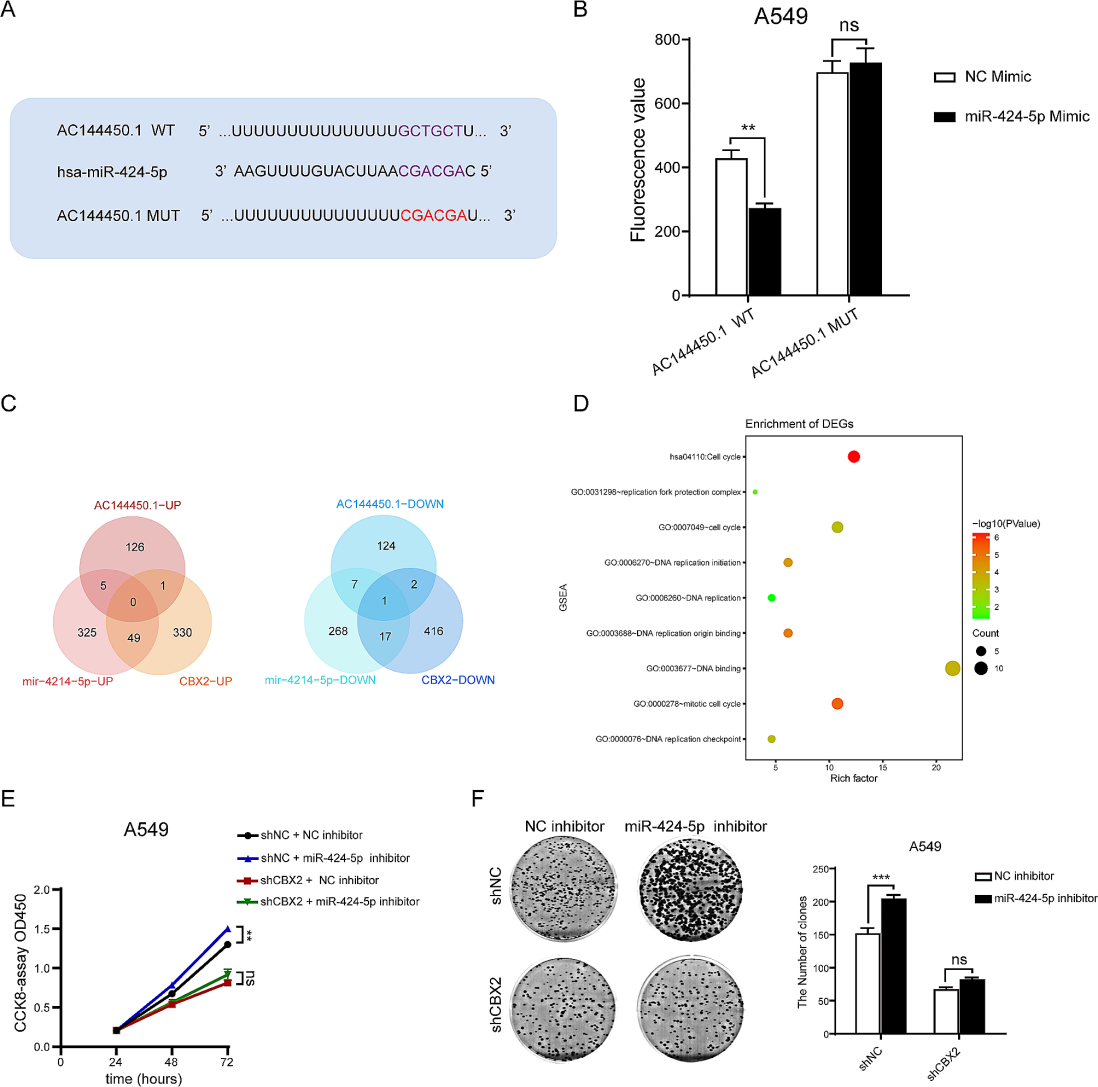
LncRNA-AC144450.1, as the ceRNA of miR-424-5p, regulated the CBX2 protein and affected the malignant progression of lung cancer. (**A**) Predicted binding site between miR-424-5p and lncRNA-AC144450.1. (**B**) Luciferase assays were performed to test the effect of miR-424-5p on wild-type or. mutant lncRNA-AC144450.1 after treating with miR-424-5p mimic. (**C**) Venn diagram showed the overlapping genes of lncRNA-AC144450.1 groups and miR-424-5p groups. (**D**) Pathway enrichment of genes co-regulated by lncRNA-AC144450.1 and miR-424-5p. (**E**) The CCK8 results of A549 cells with knockdown AC144450.1 treated with NC inhibitor or miR-424-5p inhibitor. (**F**) The clone formation results of A549 cells with knockdown AC144450.1 treated with NC inhibitor or miR-424-5p inhibitor

A complementary mutation was induced in AC144450.1 and the binding site of miR-424-5p was predicted to assess whether AC144450.1 is the ceRNA of miR-424-5p (Fig. 8A). Dual-luciferase reporter assay was used to detect changes in luciferase activity caused by changes in miR-424-5p levels before and after AC144450.1 mutation induced. The results revealed that miR-424-5p regulatedthe luciferase activity of WT AC144450.1 but not that of mutant AC144450.1 (Fig. 8B and Supplementary Fig. 8D). Thereafter, we predicted the enrichment of genes co-regulated by two or more genes in the AC144450.1/miR-424-5p/CBX2 axis on the lung cancer malignant progression pathway (Fig. 8C). The intersection genes of miR-424-5p and CBX2 were significantly enriched in the proliferation-related pathway (Fig. 8D). To validate this finding, CCK8 and clone formation assays were performed to examine cell proliferation after inhibiting miR-424-5p in shNC or shCBX2 A549 cells. The results showed that the proliferation was activated by miR-424-5p inhibitor in shNC group. However, when CBX2 was knocked down with shRNAs, the effects of the miR-424-5p inhibitor on proliferation were rescued (Fig. 8E-F and Supplementary Fig. 8E-F). Altogether, these results suggest that miR-424-5p acts as a regulator of CBX2 and affects the malignant behavior of lung cancer.

## Discussion

LUAD is a highly malignant tumor with a poor prognosis. The development of more effective diagnostic and treatment strategies for LUAD is a major focus of research at present. miRNAs are small ncRNAs that play an important role in tumor progression by regulating the cell cycle, metastasis, angiogenesis, and metabolism. They regulate gene expression and are involved in the development, progression, metastasis, diagnosis, and prognosis of cancer. For instance, the downregulation of miR-7, which has an aberrant expression pattern in lung cancer, has been demonstrated to inhibit the mRNA expression of EGFR and Raf1 [36]. Similar to miRNAs, lncRNAs play an important role in LUAD. They regulate the proliferative, invasive, and migratory capabilities of LUAD cells by interacting with miRNAs, WNT pathway, and other mechanisms [37]. The abnormal expression of lncRNAs has been associated with metastasis, advanced pathological stages, and an unfavorable prognosis among patients with LUAD. For example, dysregulated lncRNAs such as ZNF503-AS1, CYP4F26P, and RP11-108M12.3 have been associated with a poor prognosis in LUAD [38]. Therefore, lncRNAs may serve as potential targets for developing novel therapeutic strategies for LUAD. ceRNAs represent a unique mechanism of gene regulation and play an important role in the physiology and pathogenesis of diseases, including cancer. ceRNA networks of mRNAs, lncRNAs, and miRNAs have been identified in LUAD [15]. These networks regulate mRNA expression and interactions among the marker genes of tumor-associated macrophages. In this study, we identified a ceRNA regulatory network in LUAD and verified that AC144450.1, a ceRNA of miR-424-5P, regulates the malignant progression of LUAD cells. The findings of this study add to the existing knowledge of the ceRNA regulatory network in LUAD.

Cuproptosis is a copper-dependent cell death mechanism that differs from other existing cell death mechanisms. It is closely associated with cellular metabolism. Specific cancer types often exhibit elevated aerobic respiration. Studies have extensively investigated the relationship between cuproptosis-related genes and diverse tumor characteristics in multiple cancers. These studies have revealed the significant role of cuproptosis-related genes in signaling pathways associated with tumorigenesis [23]. For treating early lung cancer, cuproptosis could potentially be a valuable therapeutic approach [39]. Research into the role of cuproptosis in the treatment of early-stage lung cancer has focused on understanding the mechanisms of this form of cell death and developing prognostic models based on cuproptosis-related biomarkers [40]. Due to the significant impact of cuproptosis-related genes and pathways on mitochondrial function, researchers have focused on analyzing the prognostic value through bioinformatics and constructing prognostic models to achieve early diagnosis at the gene level [41]. A study explored the role of 16-cuproptosis-related long non-coding RNAs in predicting the clinical prognosis of patients with lung adenocarcinoma [42]. Further research on cuproptosis holds promise in enhancing our understanding of cancer and guiding clinical interventions. A notable application of cuproptosis in tumor diagnosis and treatment is the construction of risk or prognostic models [43]. The relationship between cuproptosis and LUAD has attracted substantial interest, and researchers have used online databases to identify genes involved in this relationship [44]. LUAD subtypes associated with cuproptosis have been identified and associated with clinicopathological characteristics, prognosis, and biological pathways [45]. In a study, weighted gene co-expression network analysis was used to establish a cuproptosis-based risk model for predicting the prognosis of LUAD [46]. Cuproptosis-related genes play an essential role in diagnosing and treating LUAD. In this study, the LUAD samples were uniformly clustered based on the expression of 13 cuproptosis-related genes. Patients with LUAD were divided into two groups based on the levels of cuproptosis, and differential expression analysis was performed to identify significant cuproptosis-related genes associated with the prognosis of LUAD. Altogether, the results of this study provide a potential therapeutic target for LUAD.

The CBX2 gene is responsible for maintaining the transcriptionally repressive state of numerous genes, which is crucial for regulating transcription and negatively modulating cell differentiation. CBX2 regulates the expression of various genes involved in embryonic development, sexual determination, stem cell differentiation, and primary sexual characteristics. A recent study revealed that CBX2 inhibits cancer cell proliferation and invasion by regulating the Akt/PI3K pathway [47]. Knockdown of CBX2 leads to a significant reduction in tumor cell proliferation and promotes anoikis, which increases the sensitivity of tumor cells to pods [48]. The abnormally high expression of CBX2 has been closely associated with the degree of malignancy, TNM stage, lymph node metastasis, and poor patient prognosis. Additionally, CBX2 may also influence the sensitivity of cancer cells to chemotherapeutic drugs [49]. In a study, in vitro and in vivo experiments demonstrated that knockdown of CBX2 remarkably prevented cell growth and metastasis in LUAD [35]. In addition, a similar study reported that concurrent high expression of CBX2 and EZH2 promoted the growth and metastasis of LUAD [50]. Altogether, these studies suggest that CBX2 functions as an oncogene in LUAD and serves as a promising therapeutic target for the disease. Furthermore, the involvement of CBX2 in ceRNA networks has been reported in diverse cancer types [51]. The ceRNA regulatory network involving CBX2, PBK, and AP002478.1 influences the progression of hepatocellular carcinoma [52]. CBX2 may contribute to cancer progression through ceRNA networks, suggesting that it is a promising therapeutic target for cancer. However, no studies have reported the involvement of CBX2 in ceRNA networks associated with LUAD. In this study, we established a ceRNA regulatory network involving CBX2, AC144450.1, and miR-424-5p. This network was found to be associated with the progression of LUAD. These findings provide a theoretical basis for the development of CBX2-targeted diagnostic and therapeutic strategies for LUAD.

## Conclusions

In conclusion, this study revealed that the lncRNA AC144450.1/miR-424-5p/CBX2 axis is a novel ceRNA network that regulates the malignant progression of LUAD. AC144450.1 functions as a molecular sponge of miR-424-5p and competitively regulates the expression of CBX2. In addition, the regulatory effects of miR-424-5p on the malignant progression of LUAD rely on CBX2 to a certain extent. These results provide novel insights into the complex regulatory mechanisms underlying the pathogenesis of LUAD and introduce novel targets for the diagnosis and treatment of the disease.

### Electronic supplementary material

Below is the link to the electronic supplementary material.

Supplementary Material 1

Supplementary Material 2

## Data Availability

The data generated or analysed during this study are included in this published article and any related materials are available from the corresponding author on reasonable request.

## Abbreviations

CBX2: Chromobox protein homolog 2
ceRNAs: Competitive endogenous RNAs
GSEA: Gene set enrichment analysis
lncRNAs: Long non-coding RNAs
LUAD: Lung adenocarcinoma
miRNAs: MicroRNAs
ncRNAs: Non-coding RNA
ROC: Receiver operating characteristic
UTR: Untranslated region
WT: Wild-type
