# Supplementary material for: Cuproptosis-related ceRNA axis triggers cell proliferation and cell cycle through CBX2 in lung adenocarcinoma
Source: BMC Pulm Med. 2024 Feb 14;24:85. doi: 10.1186/s12890-024-02887-0 (PMC10865584; doi:10.1186/s12890-024-02887-0)
Supplement: Supplementary file 1 — Supplementary Material 1 [file 12890_2024_2887_MOESM1_ESM.pdf]

Supplementary Figure 1

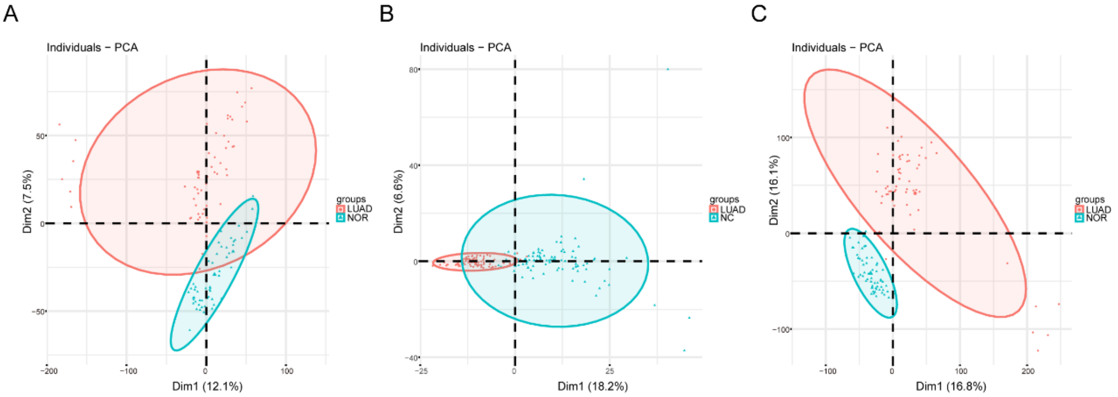

Supplementary Figure 2

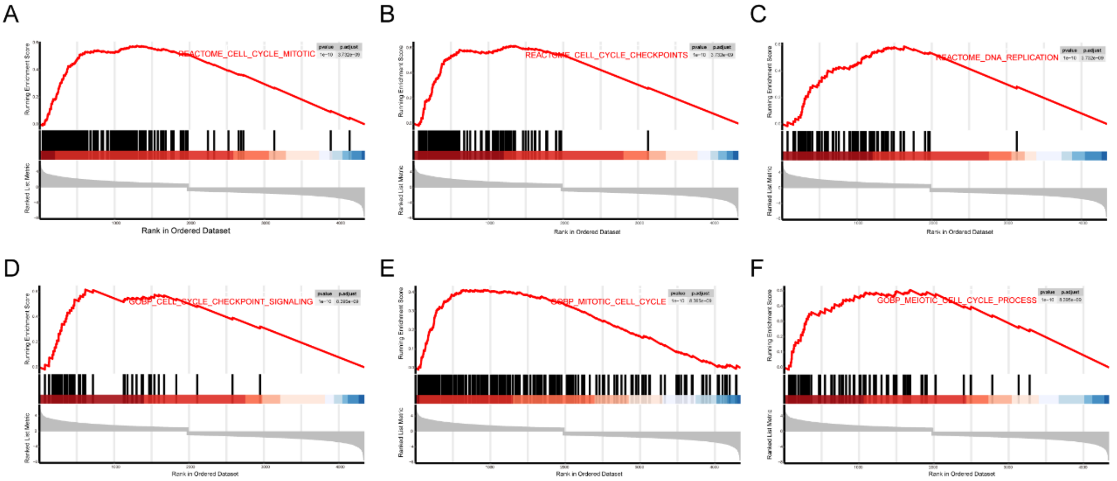

Supplementary Figure 3

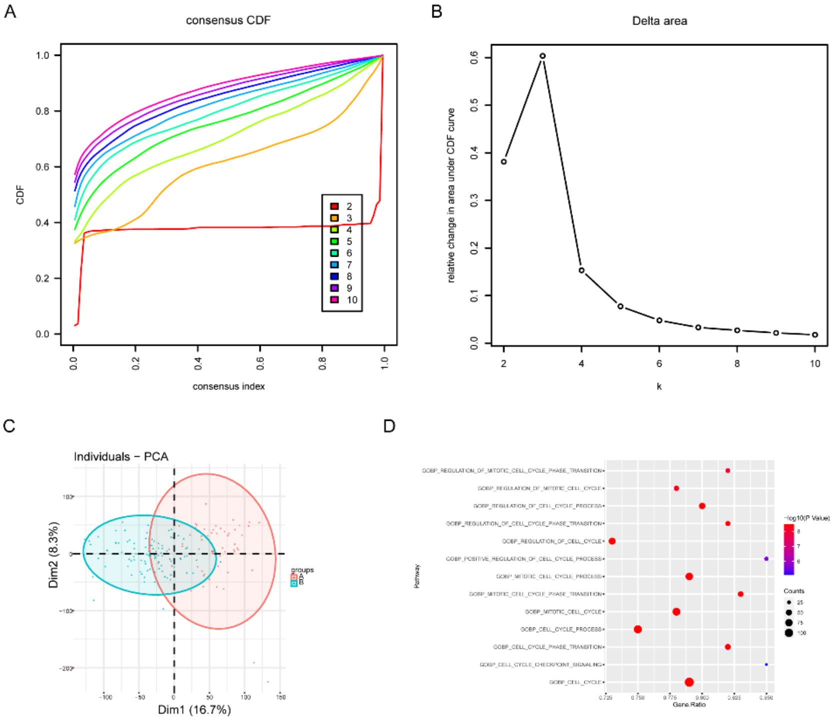

Supplementary Figure 4

A

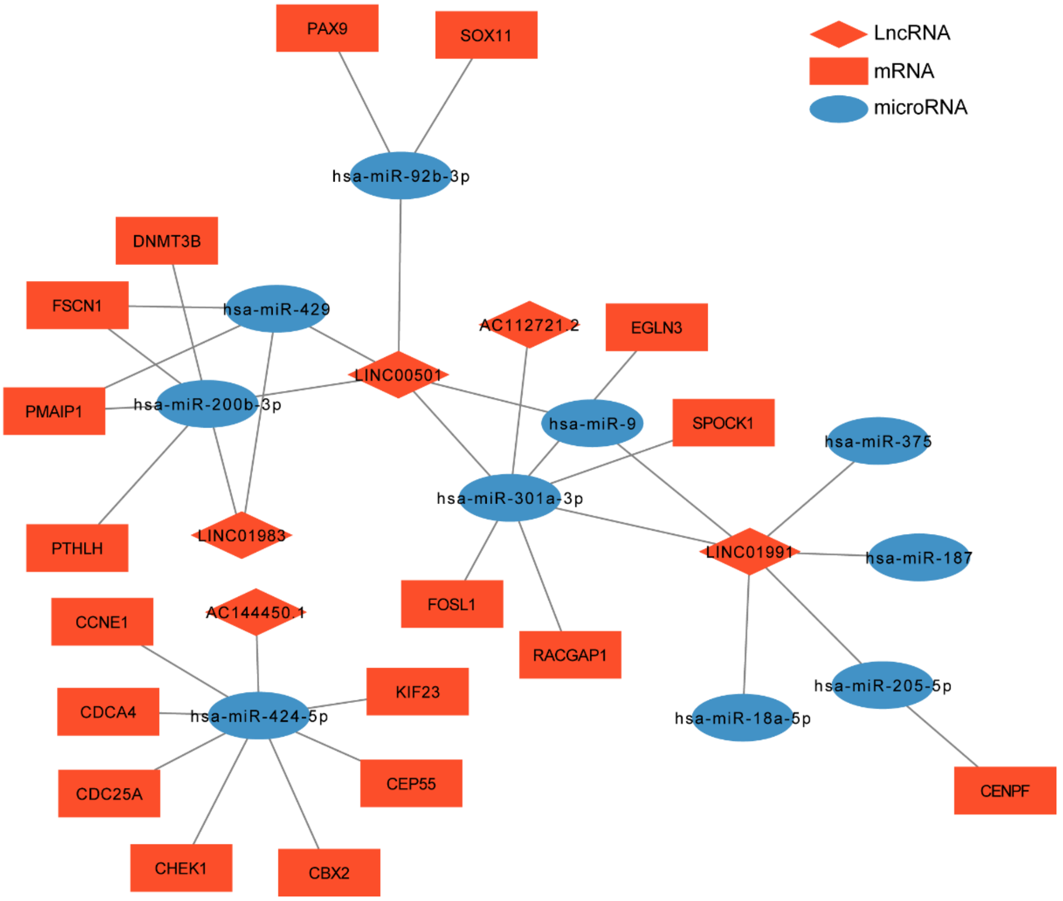

Supplementary Figure 5

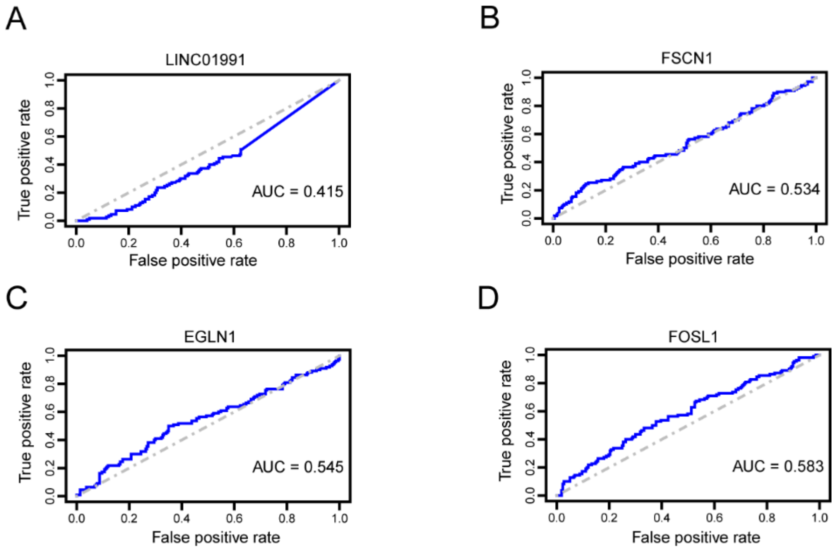

Supplementary Figure 6

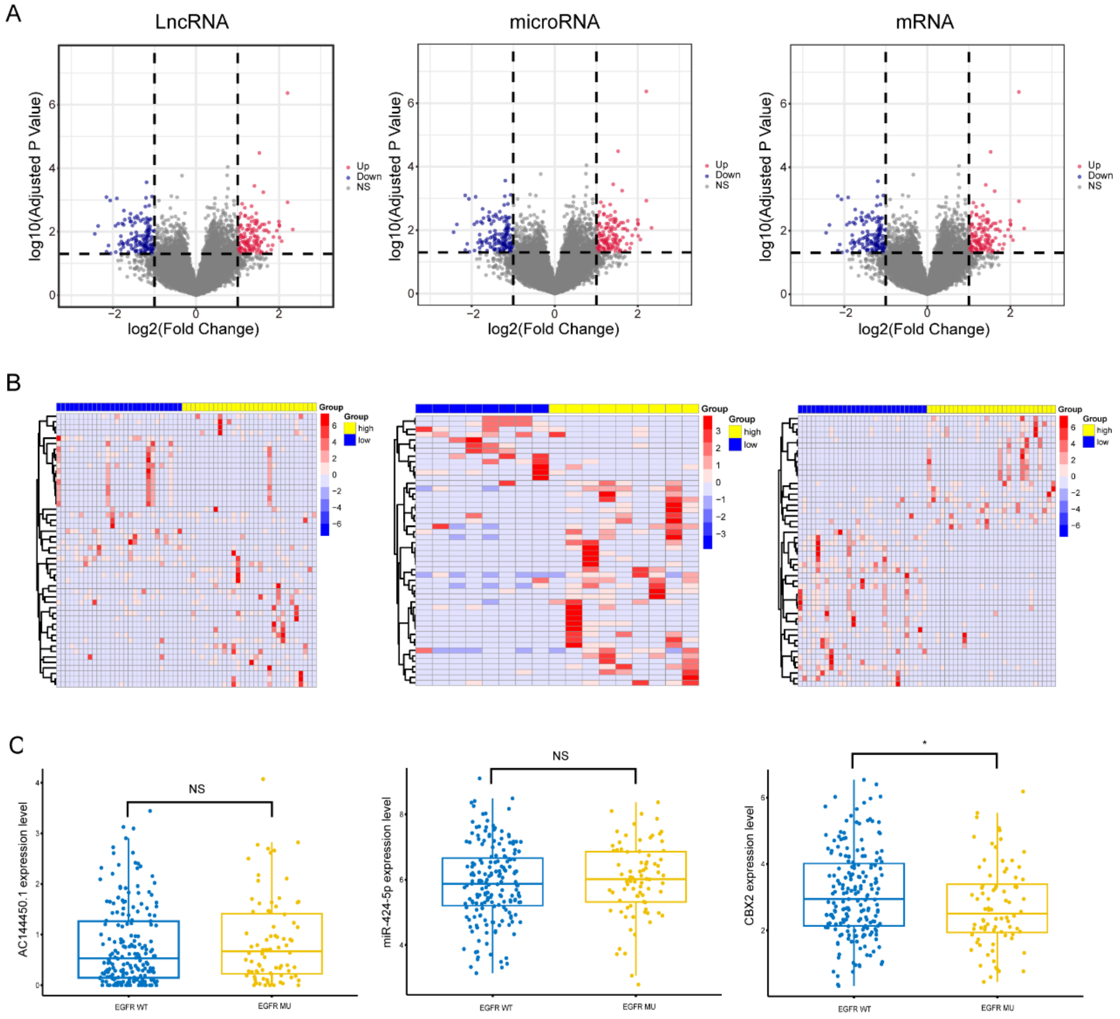

Supplementary Figure 7

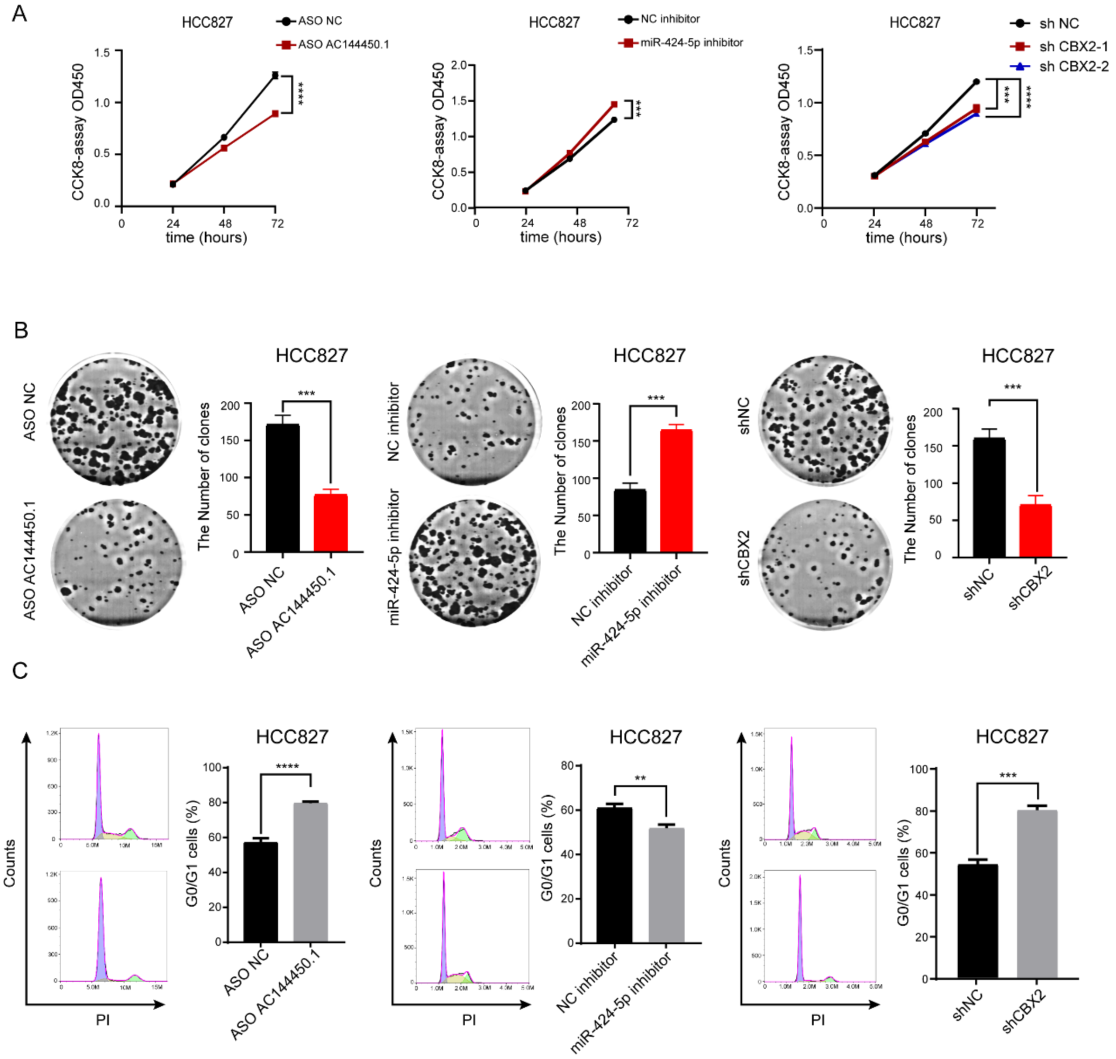

Supplementary Figure 8

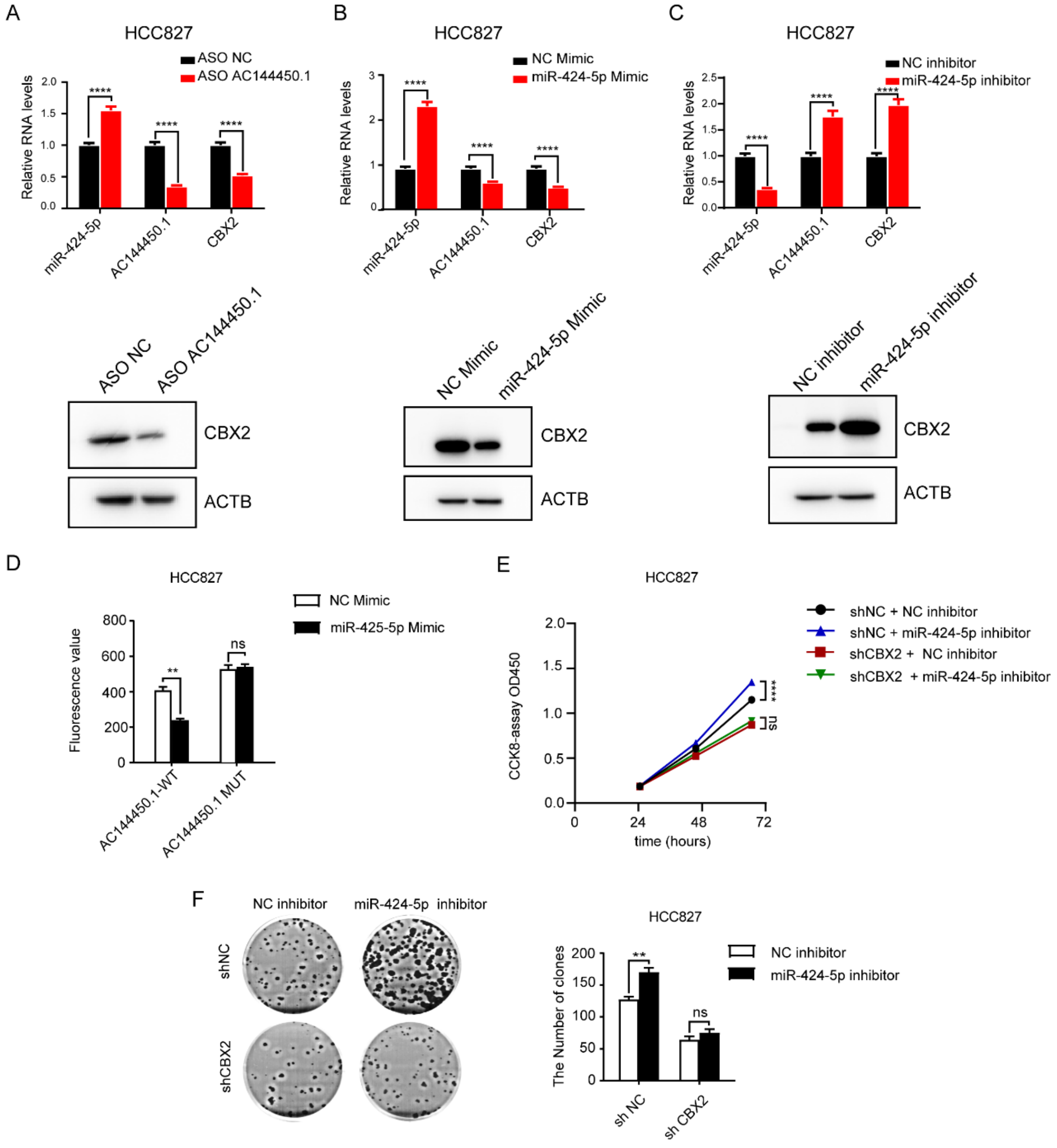

Supplementary Figure 1

(A-C) The results of differential expression analysis of mRNAs, lncRNAs and miRNAs. PCA diagrams of mRNAs, lncRNAs and miRNAs

Supplementary Figure 2

(A-F) GSEA enrichment analysis of mRNAs.

Supplementary Figure 3

(A) Cumulative distribution function (CDF).

(B) Delta area plot.

(C) PCA depicted the distribution for clusters.

(D) The bubble diagram of GO enrichment analysis of differential genes by GSEA.

Supplementary Figure 4

(A) The ceRNA network.

Supplementary Figure 5

(A-D) ROC curves of different lncRNAs.

Supplementary Figure 6

(A) The volcano map showed the differential expression analysis of high and low expression mRNAs, lncRNAs and miRNAs.

(B) The heat map showed the differential expression analysis of high and low expression mRNAs, lncRNAs and miRNAs.

(C) The expression levels of AC144450.1, miR-42 4-5p and CBX2 in EGFR wild type (EGFR WT) and EGFR mutation (EGFR MU) group of TCGA-LUAD.

Supplementary Figure 7

CCK-8 (A)、cell clone (B) and cell cycle assay (C) showed that AC144450.1, miR-424-5p or CBX2 regulate HCC827 cells malignant progression.

Supplementary Figure 8

(A) RT-qPCR assays and western blot were used to detect the expression of AC144450.1, miR-424-5p and CBX2 in HCC827 cells treated with AC144450.1 ASO.

(B) RT-qPCR assays and western blot were used to detect the expression of AC144450.1, miR-424-5p and CBX2 in HCC827 cells treated with miR-424-5p mimic.

(C) RT-qPCR assays and western blot were used to detect the expression of AC144450.1, miR-424-5p and CBX2 in HCC827 cells treated with miR-424-5p inhibitor.

(D) Luciferase assays were performed to test the effect of miR-424-5p on wild-type or mutant lncRNA-AC144450.1 after treating with miR-424-5p mimic.

(E) The CCK8 results of HCC827 cells with knockdown AC144450.1 treated with

NC inhibitor or miR-424-5p inhibitor.

(F) The clone formation results of HCC827 cells with knockdown AC144450.1 treated with NC inhibitor or miR-424-5p inhibitor.
