# Supplementary material for: Cuproptosis-related ceRNA axis triggers cell proliferation and cell cycle through CBX2 in lung adenocarcinoma
Source: BMC Pulm Med. 2024 Feb 14;24:85. doi: 10.1186/s12890-024-02887-0 (PMC10865584; doi:10.1186/s12890-024-02887-0)

**Western blot images**

Figure7A CBX2


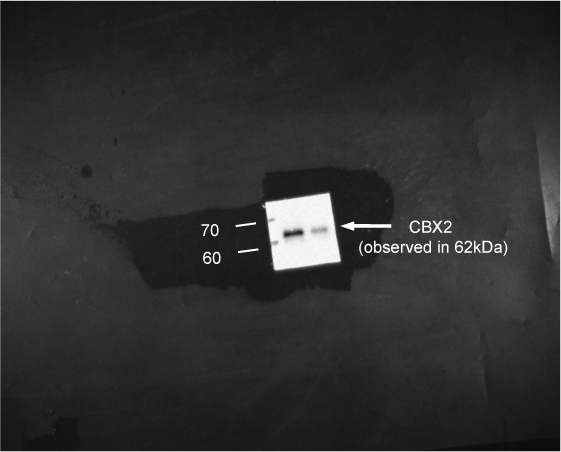


Figure7A ACTB


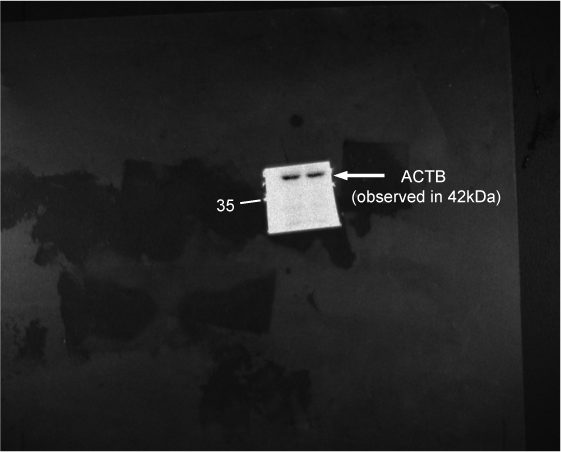


Figure7B CBX2


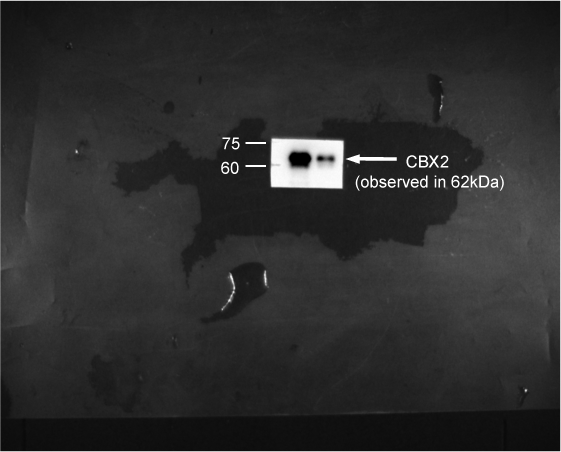


Figure7B ACTB


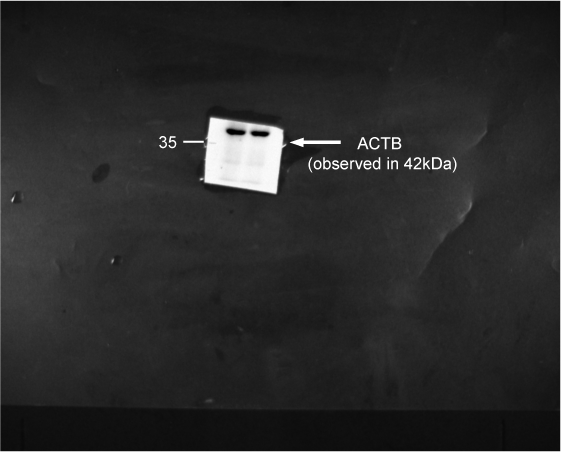


Figure7C CBX2


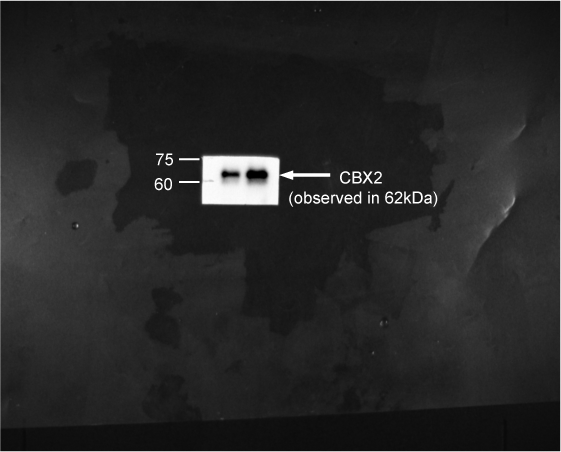


Figure7C ACTB


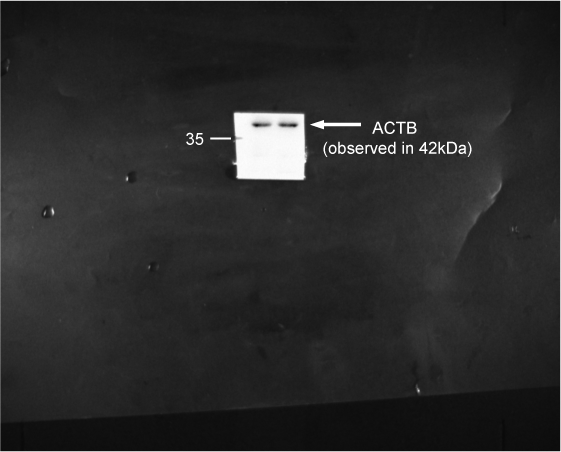


Supplementary Figure 8A CBX2


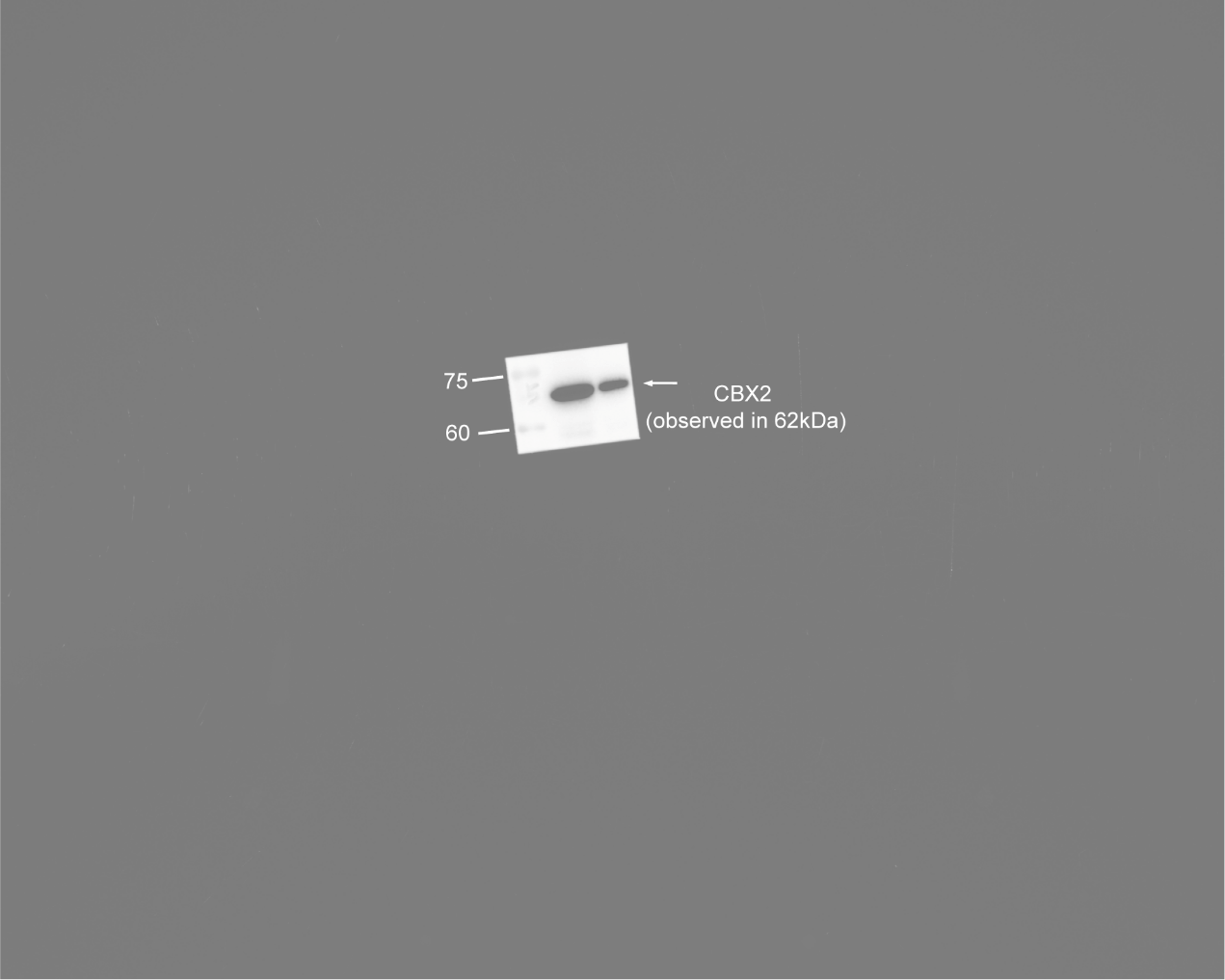


Supplementary Figure 8A ACTB


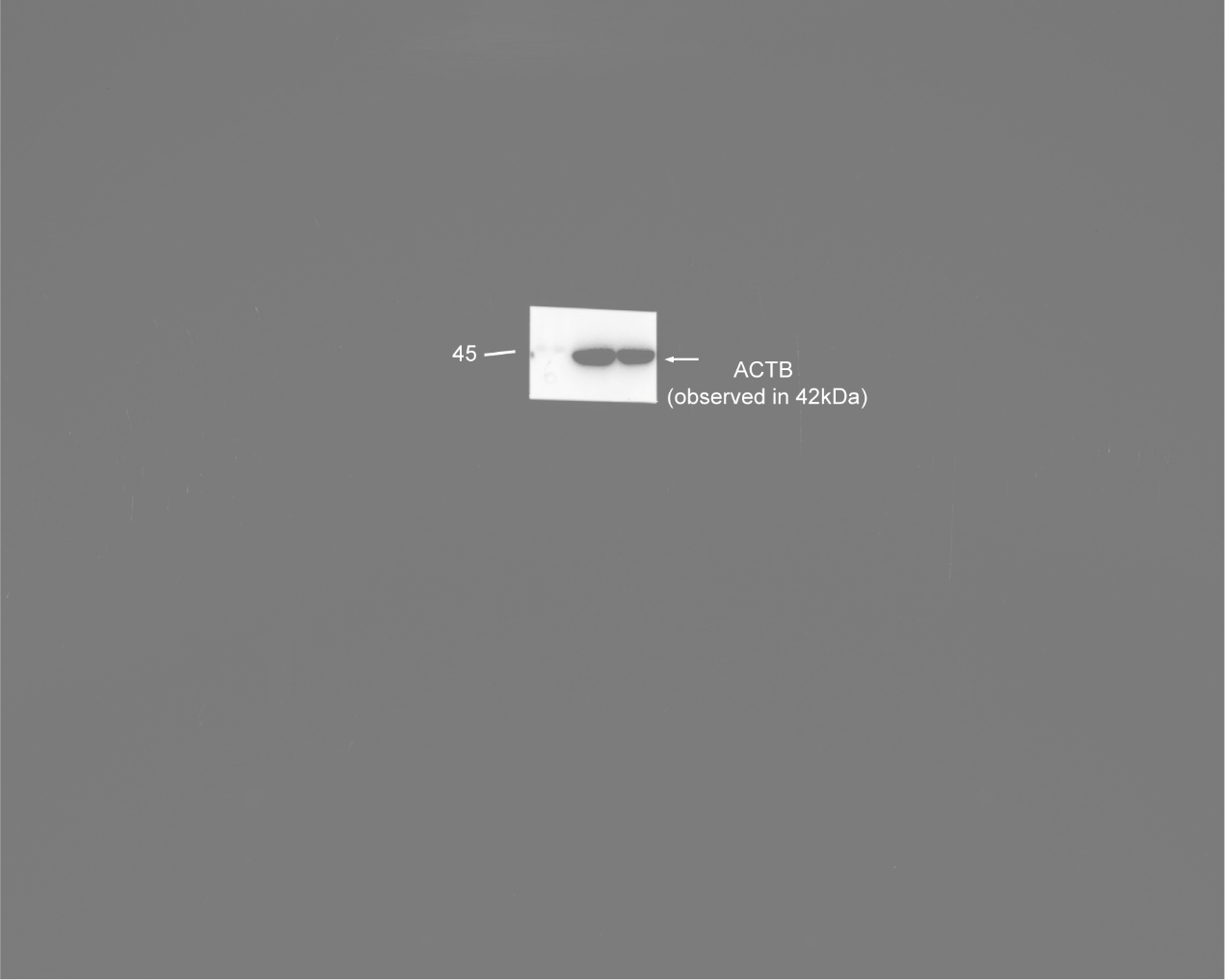


Supplementary Figure 8B CBX2


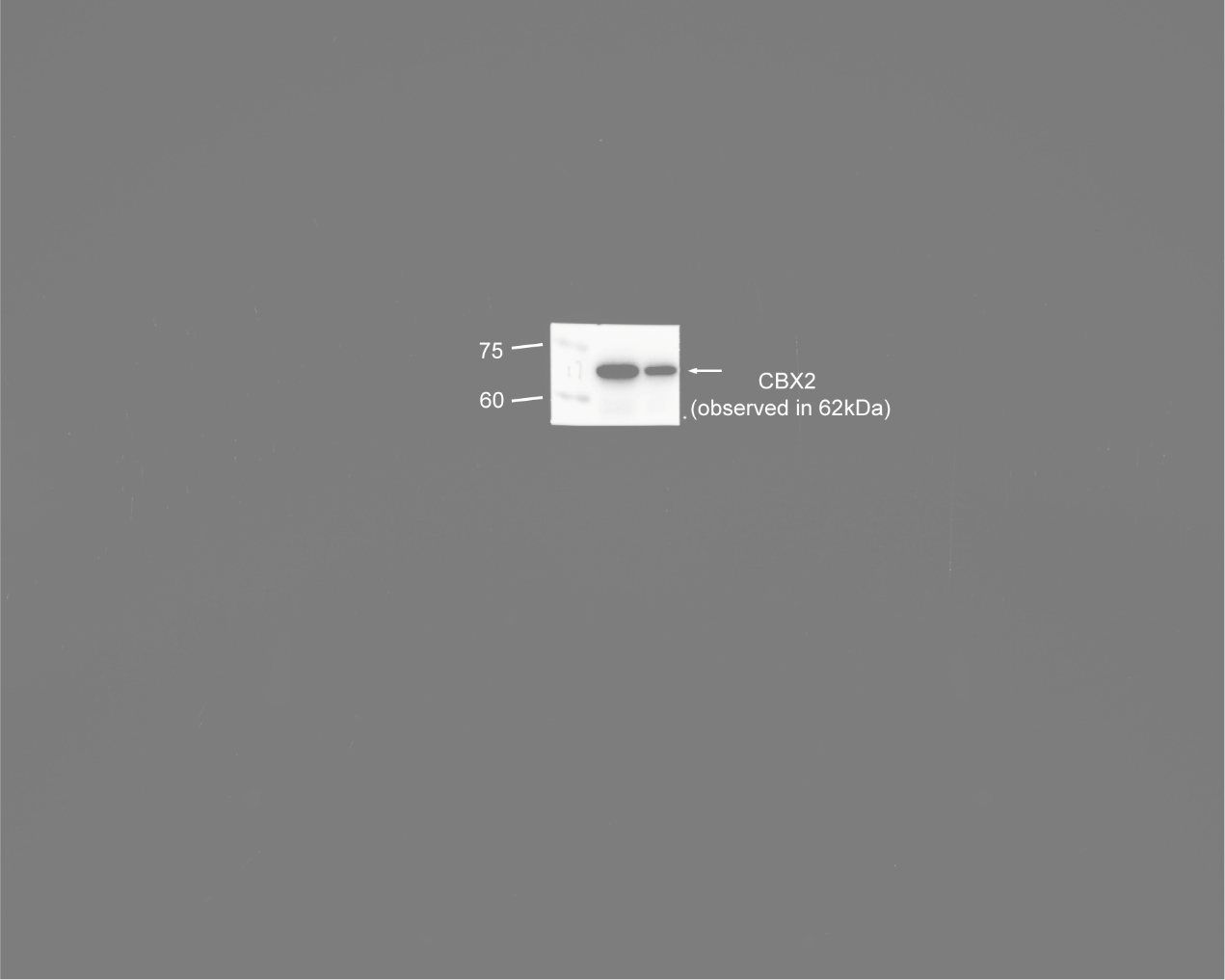


Supplementary Figure 8B ACTB


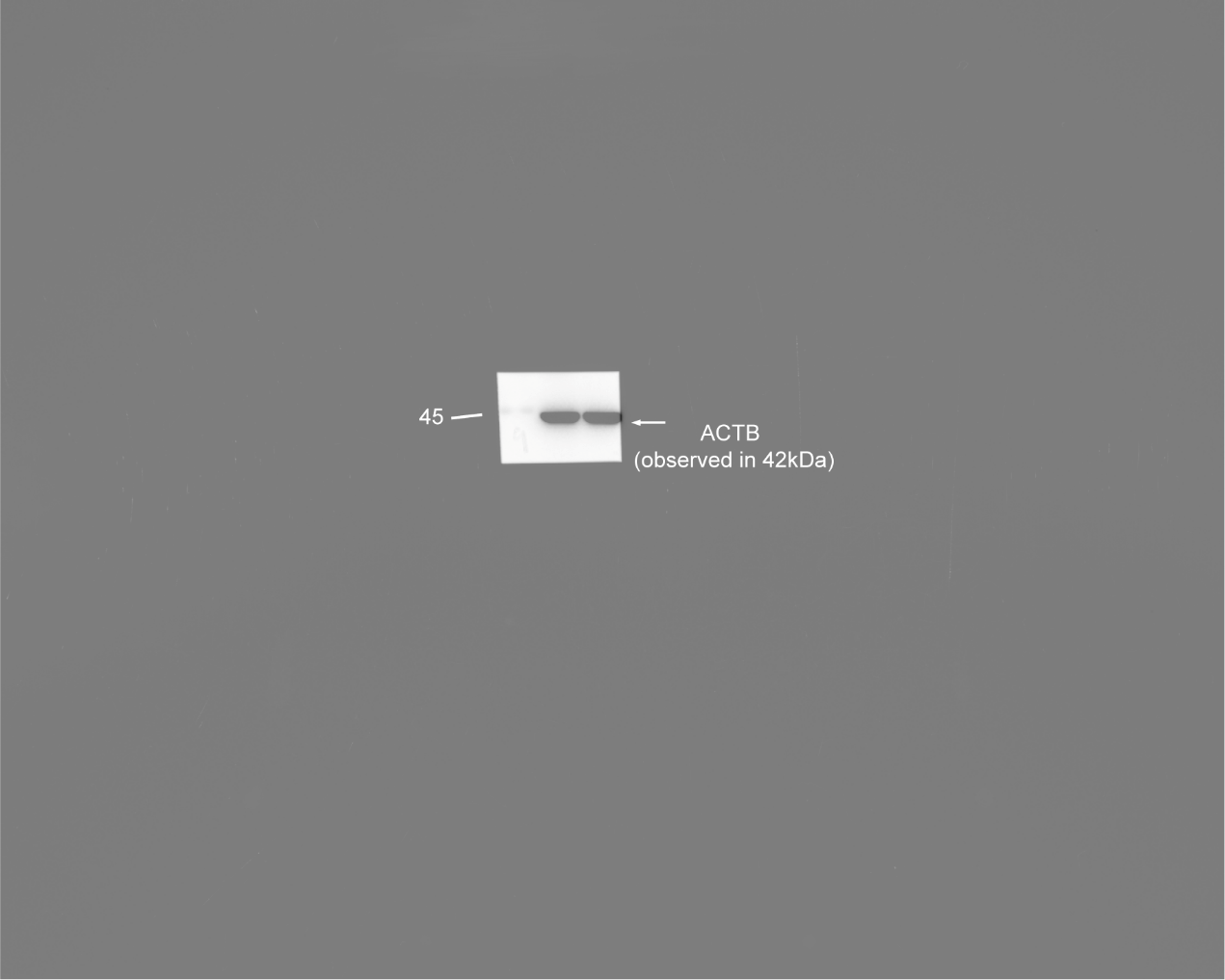


Supplementary Figure 8C CBX2


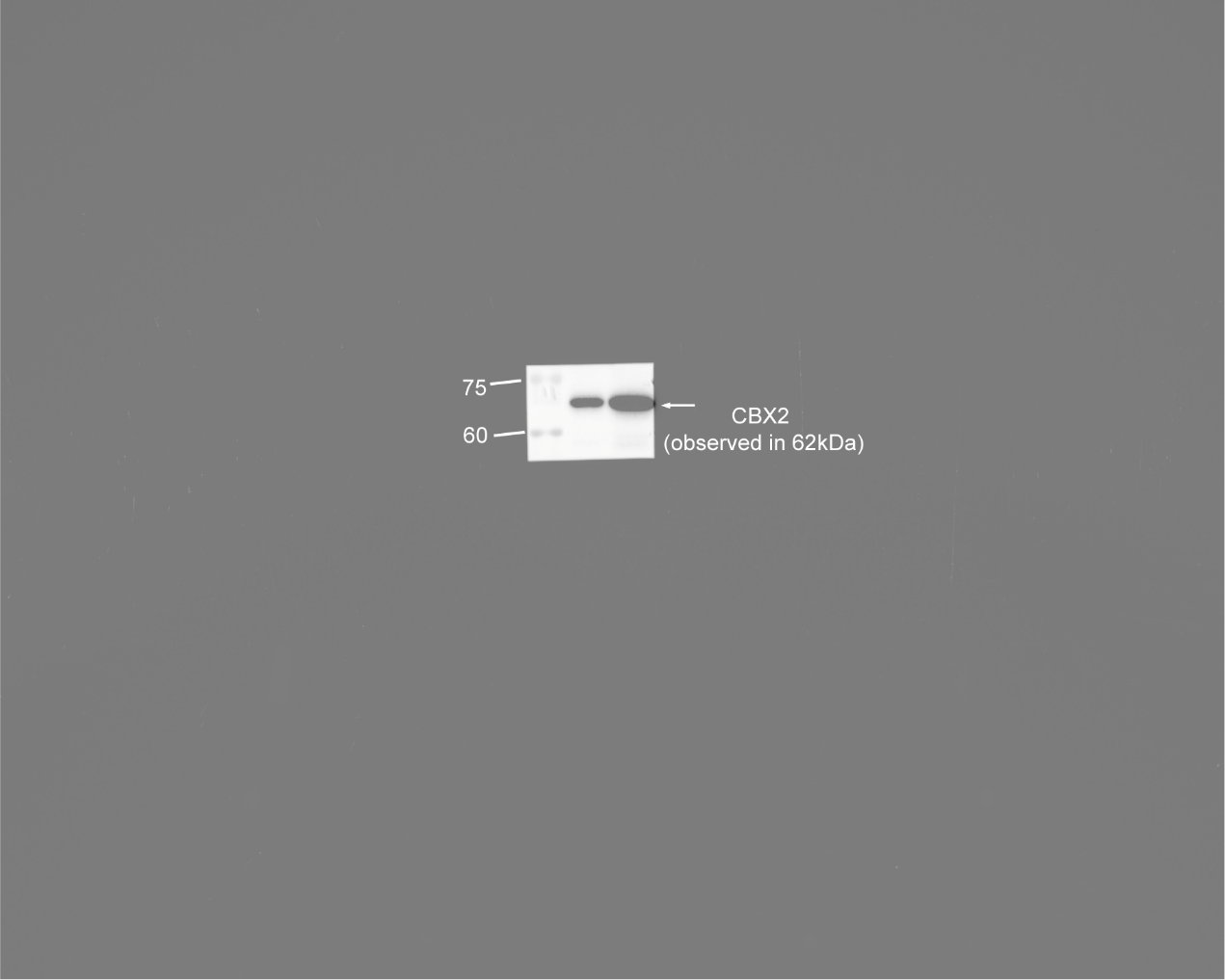


Supplementary Figure 8C ACTB


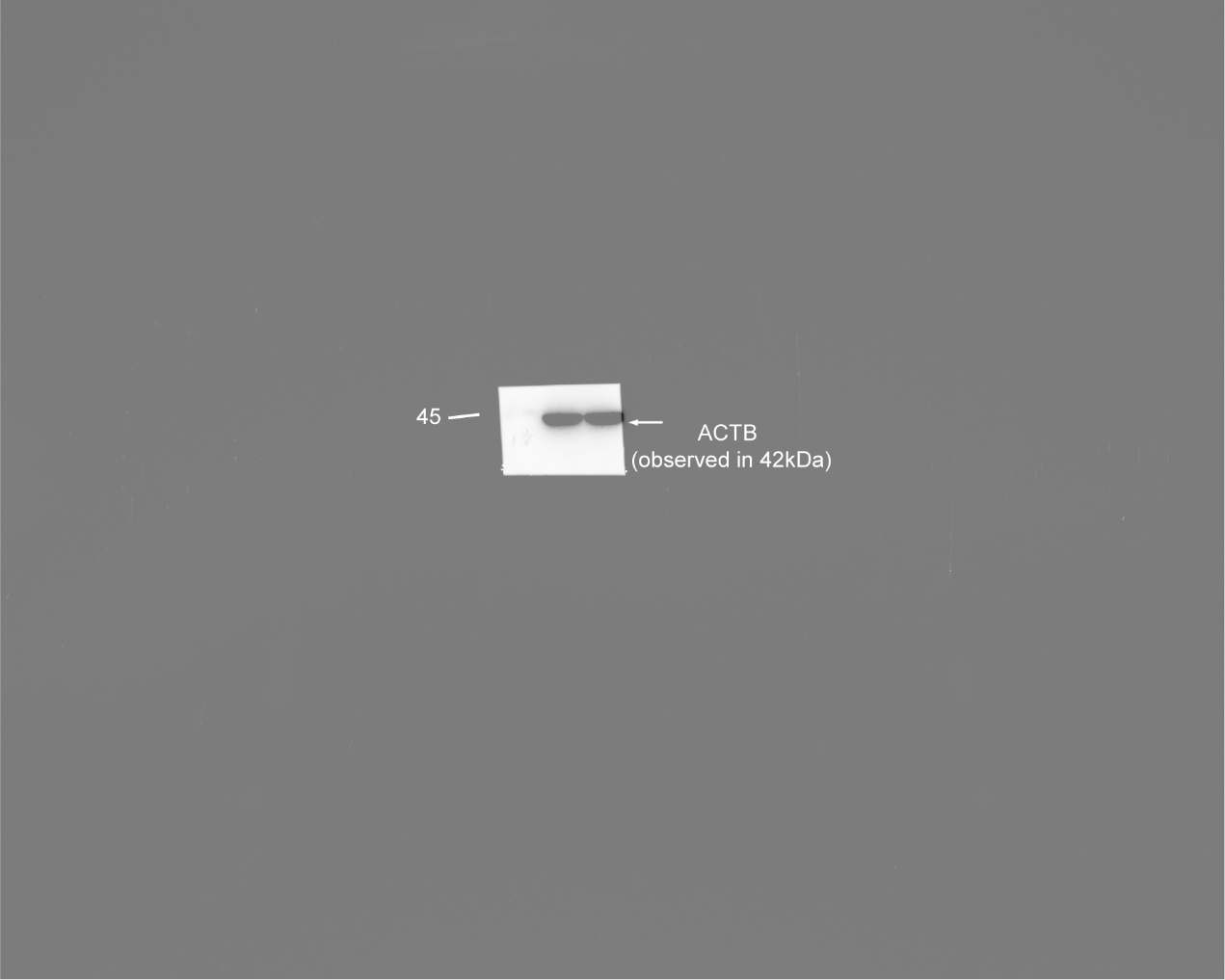

Supplement: Supplementary file 2 — Supplementary Material 2 [file 12890_2024_2887_MOESM2_ESM.docx]
